# Current Clinical and Educational Uses of Immersive Reality in Anesthesia: Narrative Review

**DOI:** 10.2196/62785

**Published:** 2025-03-11

**Authors:** Andrew Fleet, Lilia Kaustov, Elio BR Belfiore, Bill Kapralos, Clyde Matava, Julian Wiegelmann, Peter Giacobbe, Fahad Alam

**Affiliations:** 1 Department of Anesthesia Sunnybrook Health Sciences Centre Toronto, ON Canada; 2 maxSIMhealth Group Ontario Tech University Oshawa, ON Canada; 3 Department of Anesthesia and Pain Medicine The Hospital for Sick Children Toronto, ON Canada; 4 Department of Anesthesiology & Pain Medicine University of Toronto Toronto, ON Canada; 5 Department of Psychiatry Sunnybrook Health Sciences Centre University of Toronto Toronto, ON Canada; 6 Harquail Centre for Neuromodulation Sunnybrook Health Sciences Centre Toronto, ON Canada

**Keywords:** virtual reality, augmented reality, mixed reality, anesthesia, immersive reality, medical education, artificial intelligence

## Abstract

**Background:**

The concept of immersive reality (IR), an umbrella term that encompasses virtual reality, augmented reality, and mixed reality, has been established within the health care realm as a potentially valuable tool with numerous applications in both medical education and patient care.

**Objective:**

This review aimed to introduce anesthesiologists to the emerging and rapidly evolving literature on IR, its use in anesthesia education, and its transferability into the clinical context.

**Methods:**

A review of the relevant literature was conducted using the PubMed database from inception to July 5, 2023. Additional references were identified from the reference lists of selected papers.

**Results:**

A total of 51 papers related to the use of IR in anesthesia medical education (including both technical and nontechnical skills) and 63 papers related to applications in clinical practice (eg, preprocedure planning, patient education, and pain management) were included. We present evidence supporting the use of IR in the training and clinical practice of modern anesthesiologists.

**Conclusions:**

IR is useful for a variety of applications in anesthesia medical education and has potential advantages over existing simulation approaches. Similarly, IR has demonstrated potential improvements in patient care across several clinical contexts relevant to practicing anesthesiologists. However, many applications remain in the early stages of development, and robust trials are urgently needed to confirm clinical or educational effectiveness and to assess mechanisms, educational validity, and cost-effectiveness.

## Introduction

### Nomenclature and Definitions

The nomenclature surrounding immersive reality (IR) has evolved, and in this review, we provide some definitions to assist the reader. We use IR as an overarching term encompassing virtual reality (VR), augmented reality (AR), and mixed reality (MR), although other umbrella terms, such as extended reality, can also be found in the literature [1,2]. VR is any type of computer-generated immersive environment that users can interact with, including both 2D and 3D experiences. AR involves overlaying computer-generated elements on a real-world environment, and MR blends aspects of VR and AR [3,4], capturing both real-world and virtual elements in 3D and allowing for interaction across these realms [5].

IR experiences are often facilitated using a head-mounted display (HMD or headset), a piece of hardware worn on the face of the user, surrounding their field of view, with 1 or 2 small displays showing stereoscopic computer-generated imagery [6]. In this form of VR, users wearing an HMD can only perceive images of the virtually generated world displayed on the screens. This allows a user to experience a fully immersive, computer-generated 3D environment and interact with objects using handheld controllers or a set of virtual hands that mimic their own. More recently, with advances in consumer-level hardware, many IR headsets allow users to physically walk around within a defined interactive VR environment. In contrast to VR headsets, AR and MR headsets allow the user to see the real world while immersed in an overlaid computer-generated environment within the field of view of the headset.

Consumer-level HMDs most commonly used for clinical and medical education purposes incorporate variable levels of functionality (Table 1). For example, different devices are capable of tracking various types of motion. Simple systems with 3 degrees of freedom can only detect rotation around a fixed point. Newer, more complex systems, with 6 degrees of freedom can detect rotation and position in 3D space.

Regarding HMDs, 3 degrees of freedom devices can only detect where you are looking (the rotation and tilt of your head) but not your position in space (ie, whether you move forward or backward, left or right, or crouch down). Devices with 6 degrees of freedom can detect all the abovementioned types of motion using either sensors built into the device itself or external sensors, such as tripod or wall-mounted hardware.

**Table 1:** Technical characteristics and practical considerations for commonly available immersive reality head-mounted displays (HMDs) used in anesthesia.

Device	Commercial availability	Additional component requirement	HMD degrees of freedom	Eye tracking	Hand tracking	Controller degrees of freedom	Example uses in anesthesia
Oculus Rift	Discontinued	Yes, computer desktop	6 (requires external sensors)	No	No	6	VR^a^ distraction [7,8] Preprocedure education [9] Immersive VR simulation [10-12]
Oculus Go	Discontinued	No	3	No	No	3	VR distraction [13-15] Preprocedure education [16] Immersive VR simulation [17]
Oculus Quest	Discontinued	No	6	No	Yes	6	N/A^b^
Oculus Quest 2	Yes	No	6	Yes, with third-party hardware	Yes	6	N/A
Gear VR (Samsung)	Discontinued	No	3	No	No	3	VR distraction [7,18-21] Preprocedure relaxation [22]
Pico G2 4K	Yes	Yes, computer desktop	3	No	No	3	VR distraction [23]
Pico Neo 3 Pro	Yes	Yes, computer desktop	6	Yes, with additional cost	No	6	N/A
HTC Vive Pro	Discontinued	Yes, attachments	6	Yes, with third-party hardware	No	6	Immersive VR simulation [17,24-26]
HTC Vive Pro 2	Yes	Yes, attachments	6	Yes, with third-party hardware	No	6	—^c^
Valve Index	Yes	Yes, computer desktop	6	Yes, with 3rd party hardware	Yes, using controller	6	N/A
Prism Pro (Mira)	Yes	Yes, mobile device	N/A	No	No	3	N/A
PlayStation VR (Sony)	Yes	Yes, PlayStation 4 or PlayStation 5 console	6 (requires external sensors)	No	No	6	VR distraction game [27]
Google Daydream	Discontinued	Yes, mobile device	3	No	No	3	N/A
Hololens (Microsoft)	Discontinued	No	6	Yes	Yes	3	AR^d^ simulation [28]
Hololens 2 (Microsoft)	Yes	No	6	Yes	Yes	No controller	N/A
Magic Leap 1	Discontinued	No	6	Yes	Yes	6	AR simulation [29,30]
Magic Leap 2	Yes	No	6	Yes	Yes	6	N/A
Apple Vision Pro	Yes	No	6	Yes	Yes	No controller	N/A

^a^VR: virtual reality.

^b^N/A: not applicable.

^c^Data not available.

^d^AR: augmented reality.

Similar to headsets, VR controllers with 3 degrees of freedom have limited tracking ability, while newer controllers with 6 degrees of freedom allow a more naturalistic, full range of motion (eg, extending your controller in real life will be replicated in VR). Eye tracking refers to an extra set of lenses within the HMD, which tracks the movement of each eye. This allows the device to track where the user is looking and, in some cases, it allows interaction with the virtual environment through changes in gaze alone [31-33]. Hand tracking uses a front-facing camera on the HMD to track a user’s hands and project them into the virtual environment, allowing users to interact with the virtual environment without the need for controllers.

Handheld devices (eg, cell phones and tablets) can also be used for AR, by observing digital images overlaid on the real world using the camera viewer. However, these devices offer limited functionality as they have only a small field of view and are not ergonomic, as they must be held up by the user.

### The Spectrum of Immersion

Immersion is the ability of a system to create the sensation of “presence,” the subjective feeling of existing in a computer-generated world [34]. This is created in computer-based simulations by providing sensory input, including visual, auditory, and tactile (ie, haptic feedback) stimuli. Placing an individual in an IR system with these stimuli creates the sensation of being physically present in a virtual environment. Advancements in computational processing power and associated decreases in the size of electronic components have led to lower costs and rising availability of consumer-level IR technologies. This increase in availability has been accompanied by a parallel increase in the adoption of virtual-based simulation in medicine and beyond [35].

Historically, 2D worlds, which lack the increased sense of presence provided by newer 3D immersion technology, have also been labeled immersive experiences. In the context of medicine, this has mainly been limited to medical education, where learners view a 2D screen with a virtual image of a simulated task to be practiced. Many examples of simple 2D simulation exist in surgical training [36,37] and anesthesia [38-42], where students view virtual anatomical images on a screen and use partial task trainers or practical tools (eg, endoscopes, bronchoscopes, and surgical instruments), which provide haptic feedback to practice their technical skills.

Affordable HMDs and 360° cameras developed in recent years have allowed educators to increase immersion in IR educational experiences. Instead of watching on a 2D screen, learners can now be immersed in 360° experiences using headsets to experience real or animated clinical environments. As reduced work hours for medical trainees has resulted in fewer clinical learning opportunities, IR has emerged as an alternative method to supplement traditional clinical curricula. In addition, during the global COVID-19 pandemic, where social distancing and remote learning presented challenges for clinical learning, IR enabled students and residents to continue participating in interactive training. However, the literature is just beginning to examine how IR modalities can most effectively be incorporated into clinical teaching curricula.

Another relevant component of teaching curricula is simulation-based training. Simulation is an educational technique that allows interactive, immersive activity by recreating all or part of a clinical experience without exposing patients to associated risks [43]. Historically, full-body manikin simulation has been proposed as an adjunct to clinical learning, complementing clinical experience. Simulation in medical education is a well-established pedagogical practice [44], providing a viable alternative to practice with actual patients, whereby medical trainees are afforded the opportunity to train until they reach a specific competency level. Simulation ranges from decontextualized bench models and VR-based environments to high-fidelity recreations of actual operating rooms [45]. Simulation can engage the trainee in the progressive accumulation of knowledge through deliberate practice, allowing for careful matching of the complexity of the learning environment to the trainees’ current level of advancement [46]. Although simulation is widely used in medical education, it is often resource intensive, cost prohibitive [47] and requires learners to physically attend simulation centers. A survey of 154 anesthesiologists found that insufficient time and training opportunities were major barriers to taking part in simulation-based education, with 48% of respondents saying that they would be more likely to attend simulation-based courses if they were hosted at their own hospital [48].

Current advances in IR technology are addressing these limitations through the development of innovative, immersive experiences using cost-effective, consumer-level hardware capable of engaging trainees. A major advantage of IR over manikin-based simulation is increased accessibility, as learners can use the equipment on-site or access IR educational applications remotely. IR equipment can be set up in small spaces (eg, call rooms and conference rooms) with minimal time requirements so that the curricula can be delivered instantaneously in perioperative settings. In addition, although there are costs associated with developing IR applications, the end products can be made modular with the potential for both learning and assessment embedded within. IR curriculum delivery is highly cost-effective and can be easily scaled for additional learners with minimal expense, unlike manikin-based programs. These advantages may result in increased feasibility and reduced costs for IR-based simulation training, although this must still be verified using appropriate cost-benefit studies.

In addition to the use of IR simulation for education and training purposes, IR technologies are increasingly being used directly in patient care, both in patient-centric interventions (eg, as a distraction during painful procedures) and by health care providers (eg, real-time AR-image guidance). In this review, we discuss the theory underlying the use of IR technology in both medical education and clinical care as well as the evidence supporting specific examples relevant to practicing anesthesiologists.

## Methods

### Overview

This narrative review aimed to identify uses of IR relevant to practicing anesthesiologists. As such, we focused on perioperative applications and applications related to techniques in which anesthesiologists commonly take part (eg, fiber-optic intubation). Applications of IR focused on addressing pain or anxiety in instances where an anesthesiologist is not normally present (eg, burn wound dressing changes [49]) were excluded. Similarly, while a large amount of research has been done in the area of pediatric anesthesia, we chose to focus on IR applications relevant to those practicing in adult populations. IR applications in pediatric populations have been described elsewhere [50-52]. Finally, we focused on describing existing applications of IR in anesthesia, including up-to-date clinical protocols, while a review focused on theoretical and future applications has been recently published elsewhere [53].

### Search Strategy

Relevant publications were identified using PubMed and search terms including “Virtual Reality” AND (“anesthesia” OR “Anaesthesia” OR “Anesthesiology” OR “Anaesthesiology”); “Augmented Reality” AND (“anesthesia” OR “Anaesthesia” OR “Anesthesiology” OR “Anaesthesiology”); vital AND signs AND monitoring AND anesthesia AND display; (“anesth*” OR “anaesthe*”) AND (virtual reality OR immersive reality OR augmented reality OR mixed reality OR 360 video) AND (simulation) AND (education) AND (technology) AND (pedagogy OR teaching); (“anesth*” OR “anaesthe*”) AND (virtual reality OR immersive reality OR augmented reality OR mixed reality OR 360 video) AND (simulation) AND (education) AND (pedagogy OR Teaching) AND (clinical medicine); (“anesth*” OR “anaesthe*”) AND (virtual reality OR immersive reality OR augmented reality OR mixed reality OR 360 video). The literature search included articles published from inception to the date of the search, July 5, 2023. Additional references were identified from the reference lists of the selected papers.

## Results

### Education Through Immersion: Current and Potential Uses in Medical Education

#### Overview

Virtual simulation can lead to better learning outcomes for various procedures. Improved health outcomes (eg, patient safety, reduction in costs, and morbidity) have also been reported after the use of computer-enhanced training [54]. Moreover, there are many cases where both technical [55-57] and nontechnical [11] skills acquired during virtual simulation–based training have proven transferrable to subsequent performance in clinical settings.

However, studies aiming to address IR-based educational tools have been heterogeneous in their design and validation, leading to mixed results. This is compounded by the fact that many IR frameworks are designed without using educational theory as a foundation [54,58]. Literature discussing the theory behind the use of immersive technology is limited. However, by taking a broader perspective and viewing the educational literature as a whole, one can begin to form a narrative supporting its use. For example, the learning theory of constructivism outlines that learning occurs by amalgamating previous knowledge and new information to create meaning from an educational activity [59-61]. Both accuracy and real-life relevance of a problem increase learner engagement, and meaningful learning occurs when learners actively interact with the environment through decision-making. IR content is well suited to these tasks as realistic, deeply immersive clinical scenarios can be created, allowing learners to interact with their environment and to have a safe space to make mistakes, free of consequences.

Similarly, medical education has evolved toward self-directed learning and case-based practice [62-64], including the involvement of learning theories, such as self-regulated learning (SRL). SRL is a process by which learners autonomously set goals, monitor their own performance, reflect on outcomes, and then repeat the process to attain desired goals, without an emphasis on instructor-led learning and feedback [65-70]. The versatility of IR learning environments lends itself well to incorporating important components of SRL. For instance, immersive environments based on SRL concepts could provide the learner with educational objectives, allowing them to practice clinical scenarios at their own pace with mechanisms incorporated into the software to assist with progressive difficulty, automatic evaluation, and improved learning.

Many applications in health care, especially those related to nontechnical skills, are still in prototype stages that lack an explicit pedagogical basis [5]. However, this does not imply that IR applications related to clinical decision-making should not be developed. Rather, we are at a critical stage where medical learning theory must inform future IR-based learning applications. Thus, robust research studies examining the effectiveness of these applications, beyond subjective assessments, are urgently needed [71].

IR solutions have the potential to be, and in some cases already are [72,73], effective teaching tools when based on a foundation of learning theory–based design. While this foundation is not always present in current IR-based medical education solutions, we attempted to highlight examples of how IR has been used in anesthesia-based medical education for both technical and procedural teaching and nontechnical skills*.*

#### Technical Skills

Traditionally, the most common use of IR in medical education has been to train technical and procedural skills. Training programs in laparoscopic, neurological, endovascular, and orthopedic surgery have led the development of IR with positive learning outcomes [74-77]. This could be considered counterintuitive, as a principal criticism of IR in medical education has been the lack of life-like haptic (the means whereby information is conveyed through touch) technology [78], which is thought to be needed to teach technical skills.

However, educators in heavily procedural medical specialties are faced with a conundrum. On the one hand, trainees require greater autonomy as exposure to clinical settings is reduced. On the other hand, learning technical skills from text alone or by merely watching the corresponding procedure being performed has shown mixed results [79]. IR allows learners to not only visualize procedural skills in the first person but also practice the steps needed for completion in an active manner, with or without haptic capabilities. The sense of touch has been simulated using joysticks, haptic gloves, and by attaching sensors to common medical tools (eg, syringes).

Existing IR simulators with relevance to anesthesia include simulators for teaching regional anesthesia techniques [39,80-82], bronchoscopy or intubation [40,41,57,83,84], and central vein insertion [85,86] (Table 2; Table S1 in Multimedia Appendix 1) [28,38-42,56,57,80-103]. Often these applications take the form of nonimmersive VR simulators where the trainee uses a clinical tool such as a fiber-optic bronchoscope on a physical model while receiving appropriate clinical feedback (eg, ultrasound or endoscopy images) through a 2D screen. Furthermore, several MR and AR simulators have been developed that combine physical anatomical models with live tracking of handheld tools (eg, ultrasound probes and syringes) translated onto a virtual 3D computer model mimicking real anatomy [80,101,104]. Thus, the user can see, in real time, how their actions performed on the model map onto the 3D virtual anatomy model. By combining physical anatomical models and tools with IR-simulated environments, these models can effectively simulate complex procedures such as vascular access [101,104] and regional anesthesia blocks [80]. In other instances, immersive 360° VR environments have been combined with existing manikin-based simulations to increase immersion and learner engagement [91].

**Table 2:** Immersive reality (IR) interventions for both technical and nontechnical skills in anesthesia education.

Educational application				Clinical skill
**Technical skills**				
	**IR intervention**			
		AR^a^ or MR^b^ simulator (nonimmersive)	Nasogastric tube placement [96] Central line insertion [85,86,101] Intravenous insertion [90] Epidural injection [80] Transthoracic and transesophageal echocardiography [28] Lumbar puncture [87] CPR^c^ [92]	
		VR^d^ simulator (nonimmersive)	Fiber-optic bronchoscopy and intubation [40,41,57,83,84,97,99,102] Lung isolation [100] Regional anesthesia [38,39] Cystoscopy [94] Ultrasonography [98] Intravenous insertion [103] Spinal cord stimulation [42]	
		VR simulator (immersive)	Regional and spinal anesthesia [56,81] CPR and ACLS^e^ [17,88,89,91] Ultrasound guided regional anesthesia [82] Central line insertion [93]	
**Nontechnical skills (eg, clinical reasoning and communication)**				
	**IR intervention**			
		Virtual simulator (nonimmersive)	Recognition of clinical intraoperative events [105] Stress response: clinical deterioration [106]	
		AR simulator	Communication skills: ACLS scenario [29]	
		VR simulator (immersive)	Airway crisis management [26] Anesthesia crisis management and local anesthetic systemic toxicity [107] Emergency medicine [108] Operating room fire safety [10,24,30,109] Operating room infection prevention [12] Persuasion training (influenza vaccine hesitancy) [11] Preparation for an epidural procedure [110] ACLS team leadership [111] Surgical unit virtual tour [112] Therapeutic communication skills [113]	
		Multiuser VR simulator (immersive)	Operating room communication [25]	
		MR simulator (virtual humans)	Operating room communication [114] Operating room conflict resolution [115]	

^a^AR: augmented reality.

^b^MR: mixed reality.

^c^CPR: cardiopulmonary resuscitation.

^d^VR: virtual reality.

^e^ACLS: advanced cardiac life support.

#### Nontechnical Skills

IR simulations have also been adapted to teach important nontechnical skills, such as clinical reasoning, communication, and teamwork. These nontechnical skills are crucial to successful clinical encounters, and efficacious uptake of these skills is vital for reducing medical errors and enhancing patient safety. IR simulations can be used to strengthen these skills.

Immersive instructional 360° videos have been developed to teach clinical reasoning skills, allowing for more active learning by incorporating problem-solving elements into the immersive experience. For example, Masson et al [12] developed a 360° simulation that teaches perioperative infection prevention techniques and allows the learner to make choices during key decision-making points, creating a more “active” observational learning process.

Active learning can also be achieved through computer-generated immersive simulations (Table 2; Table S2 in Multimedia Appendix 1) [10-12,17,24-26,29,30,105-109,111-115]. This is especially useful for teaching comparatively rare or complex clinical events with steep learning curves (eg, airway crisis scenarios). Using commercially available HMDs (Table 1) and tracking sensors, our group [116] (NCT04451590 and NCT04591041) and others [24,26,106,111,117] created simulated crisis scenarios that allowed learners to create objectives and manage patients who are critically ill in virtual operating rooms or trauma bays. Crucially, these IR simulations respond to learners’ decisions and provide feedback upon completion. These serve as self-contained simulation scenarios learners can use for deliberate practice to supplement their clinical experiences. Specific clinical scenarios relevant to anesthesiology that have been developed include response to an operating room fire [24,118], airway crisis scenarios (NCT04451590) [26,117], advanced cardiac life support [29,111], and operating room procedures for preventing surgical site infection [12] (Table 2; Table S2 in Multimedia Appendix 1). In addition to specific clinical scenarios, IR can be used to teach and practice “soft skills,” such as teamwork and patient communication. Real et al [11] developed a simulator that uses virtual standardized patients to teach health care providers persuasion techniques for supporting patients who are hesitant about vaccines. VR training translated directly to improved clinical communication, as demonstrated by reduced vaccine refusal rates in the 3 months following VR training relative to controls [11].

In another study by Cordar et al [115], virtual humans were used as an operating room team to model conflict resolution skills during critical operating room scenarios. They found that participants were positively influenced by the virtual humans demonstrating ideal conflict resolution techniques and, conversely, were negatively influenced when they modeled negative conflict resolution behavior. These findings demonstrate that IR can be an effective method to teach nontechnical skills, such as team training and communication [115].

We have discussed the theoretical foundation for the use of IR in medical education and specific applications related to anesthesia education for both technical and nontechnical skills. IR allows learners to actively experience the complex anatomy of patients and how they interact with medical tools in a safe, accessible environment. Similarly, IR using HMDs and 360° recordings, facilitated by advances in technology, offers the ability to develop important nontechnical skills. Further information regarding the key outcomes of studies supporting the use of IR in anesthesia medical education is provided in Tables S1 and S2 in Multimedia Appendix 1. Moving forward, it is important to conduct robust trials to assess the educational efficacy of IR, determine its simulator validity, and confirm its cost-effectiveness.

### Clinical Uses of IR in Anesthesia

#### Overview

In addition to the educational applications described in the Education Through Immersion: Current and Potential Uses in Medical Education section, IR technologies are increasingly being used directly in patient care. These include patient-centered interventions, where patients are exposed to IR as an educational tool or as a distraction during painful procedures (Table 3; Table S3 in Multimedia Appendix 1) [7-9,13-16,18-23,27,32,119-144] as well as applications focused on health care providers, where IR technology and 3D visualizations are used to plan or guide clinical care (Table 4; Table S4 in Multimedia Appendix 1) [145-166]. In this Clinical Uses of IR in Anesthesia section, we discuss existing clinical uses of IR technology relevant to practicing anesthesiologists.

**Table 3:** Patient-based clinical applications of immersive reality (IR) in anesthesia.

Clinical application				Clinical scenario
**Intraprocedure distraction**				
	**IR intervention**			
		Immersive video	Awake hand surgery [13,19,139] Orthopedic surgery with regional anesthesia [7,8,138] Ambulatory surgery [144] Pregnancy termination [18] Episiotomy repair [143] Outpatient hysteroscopy [128] Lipoma excision [133] Cystoscopy [129] Bone marrow aspiration [132] Transcatheter aortic valve implantation [122] Gastrointestinal endoscopy [126] Endovascular aneurysm repair [130] Breast biopsy [134]	
		Interactive environment	Adductor canal catheter insertion [32] Upper gastrointestinal endoscopy [141,142] Cystoscopy [135] Outpatient hysteroscopy [124] Bone marrow biopsy [121] Awake hand surgery [127]	
		Interactive game	Lumbar puncture [27]	
		Guided breathing, hypnosis, or relaxation	Hip or knee arthroplasty [23,125,136] Upper limb surgery [14,15,123] Atrial fibrillation ablation [137] Epidural placement for labor [20] Endoscopic urological surgery [21]	
**Preprocedure preparation**				
	**IR intervention**			
		Operating room tour or procedural education	Elective surgery with general anesthesia [16] Elective cranial or spinal procedures [9] Colorectal cancer surgery [140]	
		Relaxation	Elective gynecological surgery [22] Cardiac surgery [131] Septorhinoplasty [119] Gastrointestinal endoscopy [120]	

**Table 4:** Applications of immersive reality (IR) focused on health care providers in clinical anesthesia practice.

Clinical application			Clinical scenario
**Clinical data visualization and vital signs monitoring**			
	**IR intervention**		
		AR^a^- or MR^b^-based HMD^c^ or smart glasses	Simulated operating room scenarios [163,165] General surgery [166] Nurse anesthetists anesthesia care [155] Supervising anesthesiologist (simulated) [156,164]
		VR^d^ HMD	Arterial blood gas monitoring [145]
**Real-time procedure guidance**			
	**IR intervention**		
		AR HMD overlay or projection	Epidural and spinal anesthesia [153,157,161] Simulated epidural and spinal anesthesia [147-149] Simulated peripheral nerve block [160,162] Simulated central venous catheterization [159] Fine-needle aspiration [158] Triage decision-making and mass casualty event [154] Percutaneous rhizotomy [146]
**Preprocedure planning**			
	**IR intervention**		
		Immersive VR HMD	Difficult airway intubation [150]
		Nonimmersive VR simulation	Regional anesthesia administration [152]
		AR or MR HMD	Awake craniotomy [151]

^a^AR: augmented reality.

^b^MR: mixed reality.

^c^HMD: head-mounted display.

^d^VR: virtual reality.

#### Clinical Data Visualization

AR-based HMDs allow users to see virtual data overlaid on the real world. For example, an anesthesiologist can view patient vital signs overlaid on the surgical field of view. Since 1995, researchers have been investigating the utility of AR-based HMDs in clinical anesthesia [166]. Several studies of anesthesiologists have reported advantages of using IR, including easier and faster recognition of clinical events [156,163,167] and the ability to spend more time focusing on the patient and the surgical field compared to vital sign monitors [168,169].

Liu et al [163] studied whether 12 anesthesiologists in a simulated environment would notice different events that occurred either on the patient monitor or in the operating room. The HMD did not interfere with participants’ ability to detect clinical events and may have allowed participants to detect some events more quickly when they were physically constrained by the need to perform a concurrent clinical procedure [163]. Conversely, Sanderson et al [164] reported that IR displays did not improve the ability to detect clinical events relative to advanced auditory displays [170], and others reported that HMDs might make individuals less likely to detect unexpected clinical events [171]. Thus, further research is needed to confirm how to optimally use HMDs, best ergonomic visualization practices, and whether increased attention leads to improved patient outcomes.

#### Preanesthesia Planning and Real-Time Guidance

Anesthesiologists often use x-rays, computerized tomography (CT), magnetic resonance imaging (MRI), and ultrasound sonography data to evaluate and plan for complex cases [172]. This provides a foundation for planning the safe management of patients with difficult airways, complex comorbidities, or complex neuraxial anatomy for regional blocks.

The use of AR-based HMDs allows for images obtained via CT, MRI, or ultrasound sonography to be mapped directly onto a patient, offering a window into the patient’s anatomy. In a surgical context, systems that generate 3D models of solid organs from CT data and overlay them onto the patient using AR enable precise planning of the optimal approach for abdominal procedures [173,174]. Similarly, our group used IR to augment real-life regional anesthesia procedures using AR holographic projections [175]. Here, live ultrasound scans are converted into holographic projections overlaid on a patient’s anatomy to serve as a pathway for an operator’s needle to follow. Holographic pathways create reliable needle trajectory guidance that has the potential to facilitate neuraxial block procedures, reducing unsuccessful attempts, needle repositioning, patient discomfort, hematomas, and failure. These studies, along with findings from simulation or phantom models [147-149], reflect the potential for IR systems in preanesthesia planning and procedure guidance, although further research is needed to confirm clinical findings [153].

#### Pain Management

IR applications have been widely studied with regard to their use as an adjunct to traditional anesthesia, analgesia, and anxiolytic techniques during painful or uncomfortable procedures [176-178]. These interventions come in many forms (eg, passive 360° videos, interactive games, guided breathing exercises, and hypnosis) and have been studied in diverse patient populations. IR applications of most relevance to anesthesiologists include distraction during minor surgery using local or regional anesthesia [7,14,15,19,125,136] as well as during epidural placement [20,123] and adductor canal catheter insertion [32] (Table 3; Table S3 in Multimedia Appendix 1).

IR is generally well received by patients and health care providers. However, studies have shown conflicting results regarding effectiveness. These contradictory findings can likely be explained by the considerable heterogeneity in study design (eg, the nature of control groups and outcome measures) and in the specific nature of the intervention (eg, passive 360° video vs interactive game) that impede useful comparisons between studies [179]. Thus, while the use of IR as a distraction technique during painful or stressful procedures is well reported [176,177], full-scale, adequately powered studies with appropriate controls are needed to confirm the effectiveness in specific patient populations.

Recent studies have shown that reductions in pain or anxiety can reduce or eliminate sedative requirements, for example, during an orthopedic surgery with spinal anesthesia [125,136] or adductor canal catheter insertion [32]. In a study of endoscopic urological surgery comparing VR distraction without sedation to sedation using intravenous midazolam, participants in the VR group required no rescue sedation and reported higher satisfaction and fewer respiratory adverse events. Despite these promising results, other recent studies have shown no difference in sedation use between IR and control groups [7,137]. Therefore, additional research is needed to clarify the observed effects and determine which populations are most likely to benefit.

Similarly, to optimize the utility of these interventions and better understand how and where they will be useful, further robust studies are needed to determine how IR is able to influence pain and anxiety. For example, are reductions in pain or anxiety the result of distraction via immersion in the virtual environment, due to the content of the intervention (eg, videos and guided relaxation), or due to the novelty of the experience itself? While distraction likely contributes to the analgesic effects of VR, some studies suggest that IR may reduce pain directly through inhibition of ascending pain pathways [180]. These findings have been verified using functional MRI studies, where reductions in brain activity secondary to pain stimuli have been demonstrated with the use of IR. Notably, improved immersion and image quality have been associated with higher levels of pain reduction [181].

#### Patient Preparation and Education

A significant proportion of patients experience anxiety before anesthesia and surgery, often triggered by fear of the unknown [182-184]. Importantly, preoperative anxiety is associated with negative patient outcomes, including increased intraoperative anesthetic requirements, increased postoperative pain, and longer recovery times [185-187]. IR offers the ability to reduce preoperative anxiety, either through immersive, guided relaxation exercises before the procedure [22] or by providing information that alleviates anxiety through exposure therapy [188,189]. In the latter case, immersive operating room tours can allow patients to witness the steps and processes leading up to the induction of anesthesia and continuing to the recovery room firsthand. While much of the research in this area focuses on pediatric populations [51,190-192], 2 randomized controlled trials have demonstrated reduced preoperative anxiety and higher patient satisfaction using VR relative to standard preoperative procedures in an adult population [9,140].

Overall, IR preprocedure preparation is well received by patients, and the creation of similar 360° content is relatively easy. This, therefore, makes for an accessible entry into IR for anesthesiologists looking to create content at low cost [193].

Further information regarding the key outcomes of studies supporting the use of IR in anesthesia-related patient care is provided in Tables S3 and S4 in Multimedia Appendix 1. However, further research is needed to assess whether these findings are reproducible and generalizable to additional patient populations.

## Discussion

### Future Applications: Practicality of the Technology and Culture Change

The continued development of low-cost, consumer-level IR technology is facilitating a shift in pedagogy from traditional 2D images and videos to learning through interactive IR environments. This is particularly important in medical education, where knowledge acquisition usually includes greater experiential, self-directed, and hands-on learning than in many other disciplines [4]. As described in this paper, IR technologies have already been used to educate trainees, prepare health care providers, and help patients. Over time, emerging technologies, such as multitouch displays, telepresence (an immersive meeting experience that offers high video and audio quality), 3D environments, natural language processing, and artificial intelligence (AI) software [194], have the potential to transform not only medical education and practice but also teaching and learning in general [195].

### Expanding Current Use

The advances in technology discussed above in the Introduction section allow for higher fidelity, increased presence, and creation of new applications in the field of IR, but it is also important to consider how existing IR technologies can extend the impact and reach of experts in the field of anesthesia. For instance, IR can be used in conjunction with traditional or virtual simulations to facilitate feedback and decrease demands on facilitator time. For example, IR can assist with the initial prebriefing [196,197], wherein participants are oriented to objectives and frameworks for their upcoming experience by facilitating a “flipped classroom” experience [198]. In this case, learners are virtually oriented to the simulation center from home, reducing the time required of facilitators and the overall time spent at the simulation center, allowing more time for participation in the simulated scenarios upon their arrival.

Similarly, telesimulation and telementoring, whereby a skilled practitioner can remotely guide a less experienced colleague through a complex procedure, either for educational purposes [199-201] or in an emergency situation [202], have been successfully used in surgical contexts [199-202]. The most complex versions of these apparatuses allow for annotations and even the movements of surgical tools, manipulated by an instructor, to be projected onto the surgical field of view of a colleague using AR technology [200,203]. This provides a proof of concept for using this technology to extend anesthesia services and aid in training anesthesiologists in underserved and rural communities, where they often work in challenging, underresourced environments to serve populations with high needs. In Ontario, Canada, the government is paying attention to the needs of such populations, especially Indigenous populations [204], as they have higher mortality rates and poorer health outcomes compared to urban populations [205]. IR applications, including telementoring and IR simulation training delivered via remote technology, are tools that can be used to advance health care in these regions and bring it in line with urban centers.

One noteworthy strength of IR over other simulation strategies is the ability to readily reimmerse in a simulation. For example, during feedback stages, IR allows both the facilitator and learner to be reimmersed in the simulation scenario. This can trigger the same emotional cues as when the learner was managing the simulated crisis, heightening recall and potentially leading to more fruitful discussions. This would similarly apply to self-debriefing, where learners are able to visualize the scenario from their own perspective but without the cognitive and emotional burden of managing the case, facilitating focus on the decision-making cues. Finally, during interprofessional training, immersive 360° cameras can be oriented to provide the point of view of each participant (eg, a surgeon could use an HMD to be immersed in the anesthesiologist’s point of view), potentially leading to a greater appreciation of one another’s roles.

The importance of well-being in health care providers has gained greater attention in recent years and presents another area where IR may have utility. Emerging evidence suggests that IR interventions, such as guided relaxation, could also help reduce stress and burnout among health care practitioners both during training [206] and in high-stress situations, such as a pandemic [207,208]. Intriguingly, a recently published small study of 20 nurses has also provided preliminary evidence that the increased usability of VR patient education compared to traditional approaches may lead to improved health care provider satisfaction and reduced burnout scores [209]. While further studies are needed, this early evidence suggests potential indirect benefits of IR, which have not been fully characterized.

### Incorporating Technological Advances

As technology advances, the ways we interact with it also evolve. Instead of interacting through touchscreens or hand controllers, we will be able to interact directly through hand motions and eye movement tracking. This may accelerate as promising design breakthroughs, such as transparent interfaces, are integrated into IR systems. AI is another emerging technology that can be incorporated into medical training and presents a potentially paradigm-shifting advancement. For instance, an early study using AI-based feedback has shown evidence for effectiveness in achieving learning goals [118]. In a simulated operating room fire scenario, this study showed a significant performance improvement when an AI-guided virtual trainer provided timely feedback to users in the virtual operating room.

Further technological advances will occur in the field of haptics. Basic haptic (touch simulation) feedback is already widely available at the consumer level through devices that provide vibration feedback (eg, video game controller “rumble packs” or mobile phone vibrations). Furthermore, pseudohaptics [210,211], where illusory haptic sensations are created using combinations of audio, visual, and kinesthetic cues, has also been used to convey various touch-based properties [212-214]. However, currently available consumer-level haptic devices cannot provide the high level of fidelity and range of motion required to simulate many tasks realistically [215], and high-fidelity haptic solutions are cumbersome, complex, and often cost prohibitive [216,217]. As consumer interest in haptics grows, haptic devices capable of providing force feedback beyond simple vibrations will become more widespread. Notably, integrating haptic feedback into IR enhances immersion by simulating sensations and has been used in medical simulations, including training and simulation of medical procedures, allowing trainees to practice realistic interactions in a safe environment.

Adopting new technological advances in IR systems benefits from the increasing availability of so-called maker spaces. These are collaborative workspaces that contain equipment such as laser cutters, computer numerical control machines, soldering irons, and 3D printers that can easily create innovative, customizable, and cost-effective learning tools. 3D printing, in particular, which is becoming widespread in part due to decreased cost, allows digitally designed objects to be replicated in the real world, facilitating the merger between IR and real physical form, albeit with some limitations [4]. In the context of anesthesia, this creates the potential for faithful recreations of anatomical features (eg, airway or spinal column) to be generated using imaging data for use in training or preprocedure planning [218,219].

### Limitations

While the potential benefits of IR simulation are considerable, the potential downsides of IR technology must also be considered. This includes the presence of cybersickness (negative symptoms associated with immersion in a digital world, including feelings of nausea, dizziness, or headache), which can limit usefulness in both educational and clinical contexts [220,221]. The incidence of cybersickness resulting from the use of IR systems depends on many different factors (eg, frame rate, field of view, sex, and previous game playing experience) and ranges from common to very rare [222]. The heterogeneity of both IR technology and interventions studied likely contributes to this variability [220]. However, both practical factors, such as limiting exposure duration, and technological factors, such as improved latency and higher levels of immersion, are known to reduce cybersickness [177,220,223], with 1 recent review concluding that benefits outweigh risks, at least in some patient populations [221]. Further research is needed to identify factors that can reduce unwanted side effects, such as cybersickness, while preserving the many advantages of IR technology.

Furthermore, it must be acknowledged that potential costs associated with the initial implementation of IR technologies, especially in low-resource settings, could be a barrier and potentially exacerbate existing inequality in health care outcomes. Until recently, IR technology was expensive, cumbersome, and restricted to several large institutions and research centers. However, recent increases in computational processing power and the accompanying decrease in the size of electronic components have led to decreasing costs and rising availability of consumer-level technologies (Table 1). The introduction of freely available game engines and mobile devices (eg, tablets and smartphones) capable of reproducing education-worthy environments with adequate fidelity has made IR technology an exciting and emerging field [4]. Widely available low-cost options, such as Google Cardboard [224] and other smartphone-based IR headsets, are suitable for many of the most common clinical uses of IR, such as patient education and pain distraction [134,225,226]. This suggests that the implementation of IR technology does not need to be limited to high-resource settings.

However, further research is needed to identify potential barriers and secondary costs related to the implementation of IR programs in different settings. Similarly, if supported by evidence from continued research, wide adoption of IR technology facilitated by funding support in low-resource settings could have an important impact on reducing inequality and generating long-term cost savings.

### From Virtual to Concrete: Are We There Yet?

Despite the great technological advancements made, particularly with respect to computational processing, it is important to consider the limitations of computer hardware with IR applications, particularly when using VR devices. More specifically, designers and developers of IR applications should consider the fidelity of their virtual environments, that is, the level to which a virtual environment resembles the real world it is meant to represent. It is not currently feasible to create a high-fidelity virtual simulation that includes all the senses (ie, input related to sight, sound, and touch). Furthermore, higher-fidelity environments require greater computer processing requirements, which may not be readily available to the average computer users.

Moreover, we must consider the high variability of users who may be susceptible to motion sickness and address accessibility issues, including those related to visual impairment and other disabilities. Finally, as IR technologies in health care are relatively new, it is essential to address ethical concerns. These include the potential short- and long-term effects of virtual environment exposure and HMD use on both patients and health care providers, as well as whether VR headsets should be classified as medical devices?

### Conclusions

In this review, we have presented the theoretical basis and evidence supporting the use of IR in the training and clinical practice of modern anesthesiologists. For those interested in implementing IR technology into their own practice, many ready-made technologies exist (Table 1). The processes and techniques required to create and implement low-cost 360° videos and virtual environments using commercially available cameras and software have been described elsewhere [116,193].

Despite recent advancements and increasing interest in IR as a research field, many applications remain in the early stages of development. Robust trials are urgently needed to confirm its clinical and educational effectiveness and to assess its mechanism, educational validity, and cost-effectiveness. While the use of IR technologies may have practical applications in medical education and for a variety of clinical patient populations, it is important to ensure that enthusiasm for this new technology does not overtake available evidence and that all applications are guided by pedagogical theory and evidence-based practice. Thus, there is a unique opportunity to innovate in a promising area of research to show the validity of IR within the anesthesia specialty and demonstrate its ability to optimize learning and clinical delivery of care. Our group founded the University of Toronto’s Collaborative Human Immersive Interaction Laboratory to develop IR solutions for clinical research, medical training, and patient education. The Collaborative Human Immersive Interaction Laboratory also aims to scientifically validate IR as a tool to enhance patient care now and in the future.

## Appendix

Multimedia Appendix 1 Summary tables of educational and clinical applications of immersive reality in anesthesia.

## Abbreviations

AI: artificial intelligence
AR: augmented reality
CT: computerized tomography
HMD: head-mounted display
IR: immersive reality
MR: mixed reality
MRI: magnetic resonance imaging
SRL: self-regulated learning
VR: virtual reality
