# Supplementary material for: Current Clinical and Educational Uses of Immersive Reality in Anesthesia: Narrative Review
Source: J Med Internet Res. 2025 Mar 11;27:e62785. doi: 10.2196/62785 (PMC11937716; doi:10.2196/62785)
Supplement: Multimedia Appendix 1 [file jmir_v27i1e62785_app1.pdf]

Multimedia Appendix 1. Educational and clinical applications of immersive reality in anesthesia summary tables.

Table S1. Educational interventions – Technical skills
Overview of studies using immersive reality for anesthesia-related teaching of technical skills, including key study outcomes. 3D, 3-Dimensional; AR, Augmented Reality; CPR, Cardiopulmonary Resuscitation; RCT, Randomized Controlled Trial; VR, Virtual Reality.

Study	Intervention	Educational scenario	Key Outcome(s)
Cailleau, et al., 2023 (RCT, n=86)[1]	Non-immersive VR simulator (ORSIM)	Fiberoptic intubation	VR training on simulated difficult airways provided no benefit relative to additional VR training on simulated normal airways
Chuan, et al., 2023 (validity, n=21 novice, 15 experienced)[2]	Immersive VR simulator (Oculus Rift S)	Ultrasound guided regional anesthesia	Demonstrated initial construct validity, scores for experienced participants were significantly higher than for novices
Huang, et al., 2023 (feasibility, n=40)[3]	MR simulator (Hololens)	Lumbar puncture	Improved success rate and time for participants
Kim, et al., 2023 (RCT, n=20)[4]	Immersive VR simulator (Oculus Quest 2)	Spinal anesthesia (Lumbar transforaminal epidural block)	VR group showed improved global rating score, procedural time and overall satisfaction relative to control (video/written materials)
Peek, et al., 2023 (RCT, n=31)[5]	Immersive VR simulator (headset not specified)	ACLS	Primary outcomes (time to reach specific clinical endpoints) were significantly worse in VR group.
Moll-Khosrawi, et al., 2022 (RCT, n=88)[6]	Immersive VR simulator (VIREED MED)	CPR (Basic Life Support)	Significantly improved outcomes (lower no flow time and overall performance) in VR group
Rochlen, et al., 2022 (observational, n=62)[7]	MR simulator (HoloLens 1)	Peripheral IV catheter placement	Demonstrated usability and positive user feedback.
White & Jung, 2022 (pre-post test, n=14)[8]	Non-Immersive VR simulator (SPINE Mentor)	Spinal cord stimulator placement	VR simulator led to improved confidence and performance scores compared to pre-test
Issleib, et al., 2021 (RCT, n=56 VR, 104 Control)[9]	Immersive VR simulator (+ CPR manikin) (headset not specified)	CPR (basic life support)	No flow time (primary outcome), an indicator of technical skill level, was improved in the control group relative to VR.
Lee, et al., 2021 (RCT Protocol, n=154 planned)[10]	MR simulator (HTC Vive Pro and CPR manikin)	CPR (Basic Life Support)	Primary outcome is mean compression depth

Mah, et al., 2021 (RCT, n=32)[11]	Immersive VR Video (Samsung Gear VR)	Central venous catheter insertion	No change in procedural time or scores with immersive video compared to standard video instruction.
Bube, et al., 2020 (RCT, n=32)[12]	Non-Immersive VR simulator	Cystoscopy	No difference in clinical outcomes (cystoscopy on two patients 3 weeks after training) between traditional and self-directed training (both groups used VR simulator)<br>Only 32% of participants performed acceptable cystoscopy after training
Nas, et al., 2020 (RCT, n=381)[13]	Immersive VR simulator (Zeiss VR One Plus)	CPR	VR group had inferior performance compared to face-to-face training
Ameri, et al., 2019 (preliminary, n=5)[14]	Non-immersive 3D AR simulator	Ultrasound-guided epidural injection	AR training had a higher success rate than using ultrasound only using a phantom trainer
Sappenfield, et al., 2019 (non-randomized, n=76)[15]	MR simulator (3D printed mannequin + emulated ultrasound imaging + instruments tracked via magnetic sensors)	Supraclavicular central vein access	Following MR training, participants were able to use this new technique to access the central vein in less attempts and shorter times compared to the standard approach
Aebersold, et al., 2018 (RCT, n=69)[16]	Non-immersive AR training via mobile device	Nasogastric tube placement	AR was well received and led to improved technical skills compared to traditional didactic teaching
Casso, et al., 2019 (Validity, n=22)[17]	Non-immersive VR simulator	Bronchoscopy	Generally positive face validity results following testing by senior anesthesiologists, although some concerns were noted
Jensen, et al., 2018 (observational, n=25)[18]	Non-immersive VR simulator	Point-of-care ultrasonography	23 out of 25 novice practitioners attained mastery in a median time of 1h 46m
Jiang, et al., 2018 (RCT, n=46)[19]	Non-Immersive VR simulator vs. high-fidelity mannequin training	Fibreoptic intubation	No difference in procedure outcomes between groups

Wong, et al., 2019 (RCT, n=34)[20]	Non-Immersive VR simulator	Fibreoptic intubation	VR group showed improved skills, following training, while the control group did not.
Rochlen, et al., 2017 (feasibility, n=40)[21]	AR simulator (mannequin with projected anatomical landmarks) (Epson Moverio BT-200 Smart Glasses)	Central line insertion	Positive feedback from participants regarding usability and utility
Mahmood, et al., 2018 (feasibility)[22]	AR simulator (Hololens)	Transthoracic and transesophageal echocardiography	Design features discussed, and opportunities for improvement identified
Latif, et al., 2016 (observational, n=15)[23]	Non-Immersive VR simulator (2x)	Fibreoptic bronchoscopy and intubation	Novice learners could be trained to proficiency (relative to practicing anesthiologists) using simulator
Nilsson, et al., 2015 (RCT, n=23)[24]	Non-Immersive VR simulator	Fibreoptic intubation	Positive training effects were observed in both groups (both groups used VR simulator, no difference between partial-task and whole-task training)
Failor, et al., 2014 (feasibility, n=13)[25]	Non-immersive VR simulator	Lung isolation	Participants rated training highly and had increased procedural confidence after training
O'Sullivan, et al., 2014 (Pilot, n=10)[26]	Non-Immersive VR simulator	Ultrasound-guided axillary brachial plexus block	No difference between standard training with our without additional VR simulation training
Robinson, et al., 2014 (pre-post test, n=54)[27]	MR simulator (3D-printed model with 3D virtual model underlaid)	Subclavian venous access	Residents showed improvements in procedural skills following training
De Oliveira, et al., 2013 (RCT, n=20)[28]	Non-Immersive VR simulator (tablet-based)	Fibreoptic intubation	Fewer failed attempts and continued improvement with simulation relative to no additional training
Kulcsar, et al., 2013 (RCT, n=27)[29]	Immersive VR simulator	Spinal anesthesia/dural puncture	VR group performed better when performing supervised procedures in clinic
Grottke, et al., 2009 (feasibility, n=5)[30]	Non-immersive VR simulator	Regional anesthesia	Discussion of regional anesthesia VR simulator capable of integrating personalized patient models

Goldmann, et al., 2006 (Observational, n=11 novices, 4 experienced)[31]	Non-Immersive VR simulator	Fibreoptic intubation	Novices trained with simulator performed faster than those without (and similar to experienced anesthesiologists)
Reznek, et al., 2002 (validity, n=41)[32]	Non-immersive VR simulator	Intravenous insertion	VR simulator demonstrated adequate validity and was appealing to users

Table S2. Educational interventions – Non-technical skills

Overview of studies using immersive reality for anesthesia-related education of non-technical skills, including key outcomes. AR, Augmented Reality; CPR, Cardiopulmonary Resuscitation; IPC, Infection Prevention and Control; RCT, Randomized Controlled Trial; VR, Virtual Reality.

Study	Intervention	Educational scenario	Key Outcome(s)
Hoek, et al., 2023 (qualitative, n=10)[33]	Immersive VR video (Oculus Go)	Therapeutic communication skills	Qualitative feedback on different communication styles and participant reflection was encouraged
Hoxbro Knudsen, et al., 2023 (validity, n=61)[34]	Immersive VR simulator (Oculus Quest 1 and 2)	Emergency medicine	Construct validity established using experienced, intermediate and novice learners.
Liaw, et al., 2023 (RCT, n=120 working in teams of 4)[35]	Non-immersive VR simulator (screen-based)	Managing stress response (Clinical deterioration, sepsis)	No difference in stress response, participant confidence or performance outcomes between groups (VR vs. face-to-face conventional simulation)
Speidel, et al., 2023 (RCT, n=61)[36]	Immersive VR video (HTC Vive)	Surgical unit virtual tour	VR 360 video effectively conveys spatial orientation and factual information simultaneously in the same learning scenario
Hess, et al., 2022 (observational, n=18)[37]	AR simulator (Magic Leap One)	Communication skills (ACLS scenario)	Medical students reported the simulation was acceptable and enjoyable
Truong, et al., 2022 (Observational, n=180, including 18 anesthesiologists) [38]	Immersive VR simulator (HTC Vive)	Operating room fire	4.4% of participants responded safely to an operating room fire on the first simulation, compared to 79% after VR training
Chheang, et al., 2020 (pilot study, n=3)[39]	Multiuser immersive VR simulator (HTC Vive)	Intraoperative communication (surgeon and anesthesiologist)	System described and feedback gathered from pilot participants regarding sense of presence, accuracy, etc.
Katz, et al., 2020 (Observational, cross-over, n=23)[40]	Immersive VR simulator (non-specified Samsung and HP headsets)	Advanced cardiac life support team leadership	Control group (high-fidelity mannequin) performed better with regard to technical processes, but no difference in decision making or communication between groups.
Lerner, et al., 2020 (feasibility, n=9 groups of 2)[41]	Immersive multiuser VR simulator (HTC Vive)	Airway crisis scenario	Participants reported training as effective, small increased in post-training knowledge was observed

Masson, et al., 2020 (RCT protocol, planned n=330)[42]	Immersive VR simulator (Oculus Rift)	Operating room infection prevention measures	Primary outcome is end of term IPC exam results (VR training vs standard slide-based lecture)
Wunder, et al., 2020 (Observational, n=32)[43]	MR simulation (Magic Leap One AR)	Operating room fire	Utility and limitations of this simulation approach were established
Abbas, et al., 2019 (RCT, n=20)[44]	Immersive VR simulator (headset not specified)	Anesthesia crisis management (local anesthetic systemic toxicity)	VR group showed improvement in some metrics, but not others after training
Erlinger, et al., 2019 (RCT, n=39)[45]	Non-immersive virtual simulator (screen-based)	Recognition of clinical intraoperative events	Students using high-fidelity mannequin simulation recognized an intraoperative event faster than when using the virtual simulation
Semeraro, et al., 2019 (feasibility, n=22)[46]	Immersive VR simulator (HTC Vive and Oculus Go)	CPR (basic life support)	A blended curriculum with traditional teaching, mannequin-based simulation and VR environments was well received by both lay people and healthcare practitioners
Sankaranarayanan, et al., 2018 (RCT, n=20)[47]	Immersive VR simulator (Oculus Rift)	Operating room fire	VR group performed better in mock OR fire scenario than those with didactic teaching alone
Cordar, et al. 2017 (observational, n=53)[48]	MR simulator (Virtual humans)	Operating room communication	Communication behaviors of anesthesia residents were positively affected by positive behaviors in virtual teammates
Dorozhkin, et al., 2017 (validity, n=49)[49]	Immersive VR simulator (Oculus Rift)	Operating room fire	Face validity was successfully established, high participant rankings for usefulness and effectiveness
Real, et al., 2017 (RCT, n=45)[50]	Immersive VR simulator (Oculus Rift)	Persuasion training (pediatric influenza vaccine hesitancy)	Vaccine refusal rate was significantly lower in the 3months after training for those in the VR group.
Cordar, et al., 2015 (RCT, n=22)[51]	MR simulator (Virtual humans)	Operating room conflict resolution	Conflict resolution behaviors were positively or negatively affected by corresponding behavior in virtual teammates.

Table S3. Patient-based immersive reality interventions in anesthesia

Overview of studies using immersive reality for patient-based, clinical interventions relevant to anesthesia. HMD, Head-mounted Display; IV, Intravenous; RCT, Randomized Controlled Trial; VR, Virtual Reality.

Study	Timing	Intervention	Patient Population	Key Outcome(s)
Baytar, et al., 2023 (observational, n=40)[52]	Pre-procedure	Immersive VR video (Samsung Gear VR)	Septorhinoplasty	VR intervention reduced pre-operative anxiety
Kim, et al., 2023 (RCT, n=40)[53]	Pre-procedure	Immersive VR Video (Samsung Gear VR)	GI endoscopy	Mean pre-procedure anxiety did not differ between control and VR group
Le Du, et al., 2023 (RCT, n=126)[54]	Intra-procedure	Immersive VR environment (Samsung Gear VR)	Bone Marrow Biopsy	Pain and anxiety scores were similar between VR and Nitrous Oxide groups
Lind, et al., 2023 (RCT, n=117)[55]	Intra-procedure	Immersive 2D VR video (HappyMed)	Transcatheter aortic valve implanation	Pain and anxiety scores were similar between groups.
Lopes, et al., 2023 (observational, n= 53 VR and 46 control)[56]	Intra-procedure	Immersive VR environment (HypnoVR)	Regional anesthesia block (upper limb orthopaedic surgery)	No difference between groups in anxiety before and after block.
Pelazas-Hernandez, et al., 2023 (RCT, n=154)[57]	Intra-procedure	Immersive VR environment (Oculus Go)	Outpatient hysteroscopy	Reduced pain scores in VR group. No change in physiological outcomes.
Barry, et al., 2022 (non-randomized, n=18 VR, 2:1 with controls)[58]	Intra-procedure	Guided Relaxation (PICO G2 4K Enterprise)	Total hip or knee arthroplasty with spinal anesthesia	VR procedures used less intraoperative sedation. Postoperative outcomes were similar between groups
Boonreunya, et al., 2022 (RCT, n=96)[59]	Intra-procedure	Immersive VR video (Oculus Go)	GI Endoscopy	No difference in pain scores or physiological metrics between groups
Faruki, et al., 2022 (RCT, n=40)[60]	Intra-procedure	Immersive VR environment (Oculus Go)	Hand surgery (regional anesthesia)	Less supplemental anesthesia (propofol) was required in VR group vs. control
Fouks, et al., 2022 (RCT, n=82)[61]	Intra-procedure	Interactive VR environment (AppliedVR)	Awake, outpatient operative hysteroscopy	No change in pain scores with VR relative to standard of care

Goergen, et al., 2022 (RCT, n=159)[62]	Intra-procedure	Immersive VR video (Exos 3D VR headset)	Rigid cystoscopy under local anesthesia	VR group had reduced heart rate variability, shorter procedures and less pain in some cases.
Krish, et al., 2022 (case report, n=2)[63]	Intra-procedure	Immersive VR video (Oculus Go)	Hand surgery (awake, local anesthesia)	Positive feedback on the VR process from participants
Lame, et al., 2022 (Case report, n=1)[64]	Intra-procedure	Immersive VR video (headset not specified)	Endovascular aneurysm repair	Patient underwent a successful surgery free of complications
Rousseaux, et al., 2022 (RCT, n=100)[65]	Pre-procedure and post-procedure	Immersive VR environment +/- guided relaxation (no headset reported)	Perioperative cardiac surgery	No significant difference between groups (control, hypnosis, VR, VR + hypnosis) in pain, anxiety or opioid use
Soret, et al., 2022 (observational, n=36 – 12 VR, 24 control)[66]	Intra-procedure	Immersive VR video (Oculus Go)	Bone marrow aspiration	No difference in anxiety or pain scores between groups.
Yesilot, et al., 2022 (RCT, n=100)[67]	Intra-procedure	Immersive VR video (smartphone-based headset not specified)	Lipoma excision with local anesthesia	VR and communication intervention reduced pain during procedure
Karaman, et al., 2021 (RCT, n=60)[68]	Intra-procedure	Immersive VR video (smartphone-based headset not specified)	Fine needle aspiration breast biopsy	Post-procedure pain and average anxiety were significantly reduced in VR group.
Ledford, et al. 2021 (case report, n=1)[69]	Intra-procedure	Guided relaxation (PICO G2 4K Enterprise)	Total hip arthroplasty with neuraxial anesthesia	Patient reported positive experience
Luczak, et al., 2021 (RCT, n=100)[70]	Intra-procedure	Immersive VR environment (headset not specified)	Rigid cystoscopy under local anesthesia	VR group had lower pain scores and physiological markers, but higher levels of nausea
Peuchot, et al. 2021 (Observational study, n=20)[71]	Intra-procedure	VR hypnosis (HypnoVR)	Total knee arthroplasty with spinal anesthesia	VR patients required less sedation<br>No change in postoperative anxiety

Roxburgh, et al. 2021 (observational, n=99)[72]	Intra-procedure	Guided breathing and music (Deepsen)	Atrial fibrillation ablation with awake sedation	Pain improved in VR group<br>No change in morphine consumption
Tharion, et al., 2021 (RCT, n=90)[73]	Intra-procedure	Immersive video (Smartphone + PRO1 HMD)	Arthroscopic knee surgery with spinal anesthesia	Significantly higher patient satisfaction an no change in anxiety using VR vs IV midazolam
Touil, et al., 2021 (observational, n=48)[74]	Pre-procedure /Intra-procedure	Immersive VR/Guided relaxation (Gear VR)	Loco-regional (axillary plexus) anesthesia block for hand surgery	Pre-operative anxiety was significantly reduced after VR session.
Turrado, et al., 2021 (RCT, n=126)[75]	Pre-procedure	Immersive VR video (OR Tour) (BlueBee Genuine VR)	Colorectal cancer	Pre-operative anxiety was reduced using VR operative process tour
Vogt, et al., 2021 (RCT, n=84)[76]	Pre-procedure	Immersive video (OR tour) (Oculus Go)	Elective surgery with general anesthesia	No change in anxiety with VR, but high patient satisfaction
Alaterre, et al., 2020 (before-after study, n=100)[77]	Intra-procedure	Guided meditation (Oculus Go)	Upper limb surgery, peripheral nerve block	Higher patient satisfaction and lower intraoperative anxiety with VR
Chan, et al., 2020 (Observation study, n=108)[78]	Pre-procedure	Guided relaxation (Gear VR)	Minor elective gynecological surgery	Anxiety and depression were reduced post-intervention, relative to before
Huang, et al., 2020 (RCT, n=50)[79]	Intra-procedure	Immersive video (Gear VR or Oculus Rift DK2)	Total hip/knee arthroplasty, regional anesthesia	No difference in sedation requirements in VR group
Kist, et al., 2020 (Case report, n=3)[80]	Intra-procedure	Guided relaxation (Gear VR)	Epidural placement for labour	VR helpful for self-reported pain and anxiety
Sridhar, et al., 2020 (Pilot RCT, n=30)[81]	Intra-procedure	Immersive environment (unspecified device)	First trimester pregnancy termination	Decreased anxiety during and after procedure with VR
Faruki, et al., 2019 (RCT protocol,	Intra-procedure	Guided relaxation (Oculus Go)	Upper extremity orthopaedic surgery with peripheral nerve block	Study ongoing (primary outcome difference in propofol use)

planned n=40)[82]				
Hoxhallari, et al., 2019 (RCT, n=41)[83]	Intra-procedure	Immersive video (Gear VR)	Awake hand surgery under local anesthesia	Lower anxiety reported with VR, no change in pain scores
Moon, et al., 2018 (RCT, n=37)[84]	Intra-procedure	Guided relaxation (Gear VR)	Endoscopic urologic surgery with spinal anesthesia	VR distraction with no sedatives increased patient satisfaction and reduced respiratory side effects relative to midazolam.
Thomas, et al., 2018 (Case report, n=1)[85]	Intra-procedure	Interactive game (Playstation VR)	Lumbar puncture	Reduced sedative requirements, procedure time and recovery time relative to previous procedures in same patient
Chan, et al., 2017 (pilot study, n=19)[86]	Intra-procedure	Immersive video (Oculus Rift DK2)	Joint replacement with regional anesthesia	Sedative use was lower in the VR group
Mosso, et al., 2017 (RCT, n=115)[87,88]	Intra-procedure	Interactive environment (eMagin HMD)	Upper GI endoscopy	VR distraction reduced subjective pain scores and corresponding physiological responses
Pandya, et al., 2017 (retrospective, n=14)[89]	Intra-procedure	Interactive environment (Hypervision 2D VR glasses)	Preoperative adductor canal catheter insertion	VR group received less IV sedation and reported less pain
Bekelis, et al., 2016 (RCT, n=127)[90]	Pre-procedure	Immersive video (Peri-operative process) (unspecified Oculus device)	Elective cranial and spinal procedures	Higher satisfaction and lower anxiety observed with VR
Jahani Shoorab, et al., 2015 (RCT, n=30)[91]	Intra-procedure	Immersive video (Wrap 920, Vuzik)	Episiotomy repair	Reduced pain in VR + lidocaine group vs lidocaine alone
Mosso, et al., 2009 (RCT, n=21)[92]	Intra-procedure	Immersive video (Vuzik iWear AV 920)	Ambulatory surgery with local/regional anesthesia	Reduced anxiety in VR group

Table S4. Healthcare provider-focused clinical immersive reality interventions

Overview of studies using immersive reality for healthcare-provider-focused clinical interventions relevant to anesthesia. 3D, 3-Dimensional; AR, Augmented Reality; HMD, Head-mounted Display

Study	Use	Intervention	Clinical scenario	Key Outcome(s)
Bergauer, et al., 2023 (RCT, n=50 anesthesiologists)[93]	Vital Signs Monitoring	Immersive VR HMD (Visual Blood, Oculus Quest 2)	Arterial Blood Gas (ABG) monitoring	Correct perception of ABG parameters was similar between Visual Blood VR and standard printouts
Berger, et al., 2023 (case report, n=1)[94]	AR guidance	AR HMD (Medivis SurgicalAR)	Percutaneous rhizotomy	AR-assisted fluoroscopy guided percutaneous glycerol rhizotomy was successfully performed on 1 patient.
Hayasaka, et al., 2023 (RCT, n=30)[95]	AR guidance	AR HMD (HoloLens2)	Epidural anesthesia	Optimal needle placement was significantly improved in AR group vs control
Jun, et al., 2023 (proof-of-concept, n=1 anesthesiologist)[96]	AR guidance	AR HMD (HoloLens2)	Epidural injection (Lumbosacral transforaminal epidural injection)	Procedure duration was shorter in using AR, but no significant difference in needle accuracy
Reinacher, et al., 2023 (RCT, n=4, 20 attempts each (10 normal, 10 with AR)[97]	AR guidance	AR HMD (Magic Leap 1)	Epidural anesthesia	Success rate, distance from optimal placement and procedure time were improved with AR.
Iliff, et al., 2022 (case report, n=1)[98]	Pre-procedure planning	HMD (HTC Vive)	Difficult airway planning	CT and MRI imaging form 1 patient was visualized using VR, resembling virtual endoscopy
Moon & Barua, 2022 (usability, n=10, including 2 anesthetists)[99]	Pre-procedure planning	MR HMD (Magic Leap 1)	Awake craniotomy planning	Acceptable usability demonstrated for multi-disciplinary team
Seong, et al., 2022 (Case Report, n=3)[100]	Pre-procedure planning	Non-immersive virtual simulation	Regional anesthesia	Three challenging regional anesthesia blocks were successfully preformed using the simulation
Tanwani, et al., 2022 (proof-of-concept, n=7 anesthesia resident/staff)[101]	MR guidance	MR HMD (HoloLens1)	Ultrasound guided regional anesthesia (neuraxial)	Preliminary results suggest technique is easy for novice and experienced learners
Follmann, et al., 2021 (crossover trial, n=40)[102]	Technical Guideline presentation	AR glasses (Recon Jet)	Triage decisions in mass casualty event	AR took more time per assessment than tablet PC, but was reduced with practice. No difference in accuracy.

Romare, et al., 2021 (qualitative, n=7)[103]	Vital Signs Monitoring	HMD (Google Glass)	Nurse anesthetists administering anesthesia care	Feedback gathered and opportunities for improvement identified.
Schlosser, et al., 2019 (proof-of-concept, n=8)[104]	Vital Signs Monitoring	HMD (Vuzix M300 HWD)	Supervising anesthesiologist, simulated operating rooms	Significantly more patient alarms were noticed using the HMD
Hetherington, et al., 2017 (validity, n=20)[105]	Real Time Guidance	AR overlay (PicoPro Projector)	Ultrasound-guided epidural/spinal anesthesia	Successful identification of vertebral levels in real time
Kaneko, et al., 2017 (proof-of-concept, n=32)[106]	Real Time Guidance	AR HMD (Moverio BT-200)	Ultrasound guided fine-needle aspiration in neck	No difference in procedural outcomes (time, needle redirections), but potentially improved ergonomics (reduced number of head turns)
Kaneko, et al., 2016 (pilot study, n=5)[107]	Real Time Guidance	AR HMD (Moverio BT-200)	Simulated ultrasound-guided central venous catheterization	No difference in procedural outcomes (time, needle redirections), but potentially improved ergonomics (reduced number of head turns)
Przkora, et al., 2015 (validity, n=20)[108]	Real Time Guidance	AR HMD (Wrap, Vuzik)	Simulated ultrasound-guided peripheral nerve block	HMD improved procedure time and reduce head and probe movements
Ashab, et al., 2012 (validation, n=10)[109]	Real Time Guidance	AR overlay (MicronTracker SDK + 3D Slicer)	Ultrasound-guided epidural	AR was more successful in identifying vertebral levels than manual methods
Udani, et al., 2012 (Feasibility, n=2)[110]	Real Time Guidance	AR HMD (MicroOptical CV-3)	Simulated ultrasound-guided popliteal-sciatic nerve blocks	Confirmed feasibility of their approach
Liu, et al., 2009 (repeated measures, n=12)[111]	Vital Signs Monitoring	HMD (Microvision Nomad)	Simulated operating room scenarios	Participants were able to detect some clinical events faster using HMD, especially when physically constrained by another task. However, some events were detected more slowly.
Sanderson, et al., 2008 (repeated measures, n=16)[112]	Vital Signs Monitoring	HMD, vs advanced auditory monitoring (Sony U50 Palmtop PD)	Supervision of simulated junior anesthesia resident during simulated operation	Participants were able to detect more clinical events of interested using audio-based vital signs monitoring than HMD or traditional visual monitors
Ormerod, et al., 2003 (feasibility,	Vital Signs Monitoring	HMD (Microvision Nomad)	Anesthesiologists, simulated operating room scenario	Anesthesiologists had reduced shifts in attention with HMD,

n=unknown)[113]				allowing more time spent looking at patient
Block, et al., 1995 (feasibility, n=11)[114]	Vital Signs Monitoring	Data projected directly into one eye (“Private Eye, Reflection Technology)	Anesthesiologists, general surgical list	Generally favorable response, but technical issues were identified

References

1. Cailleau L, Geeraerts T, Minville V, Fourcade O, Fernandez T, Bazin JE, Baxter L, Athanassoglou V, Jefferson H, Sud A, Davies T, Mendonca C, Parotto M, Kurrek M. Is there a benefit for anesthesiologists of adding difficult airway scenarios for learning fiberoptic intubation skills using virtual reality training? A randomized controlled study. PLoS One 2023;18(1):e0281016. PMID:36706107

2. Chuan A, Qian J, Bogdanovych A, Kumar A, McKendrick M, McLeod G. Design and validation of a virtual reality trainer for ultrasound-guided regional anaesthesia. Anaesthesia 2023 Jun;78(6):739–746. PMID:37010989

3. Huang X, Yan Z, Gong C, Zhou Z, Xu H, Qin C, Wang Z. A mixed-reality stimulator for lumbar puncture training: a pilot study. BMC Med Educ 2023 Mar 22;23(1):178. PMID:36949483

4. Kim JY, Lee JS, Lee JH, Park YS, Cho J, Koh JC. Virtual reality simulator’s effectiveness on the spine procedure education for trainee: a randomized controlled trial. Korean J Anesthesiol 2023 Jun;76(3):213–226. PMID:36323305

5. Peek JJ, Max SA, Bakhuis W, Huig IC, Rosalia RA, Sadeghi AH, Mahtab EAF. Virtual Reality Simulator versus Conventional Advanced Life Support Training for Cardiopulmonary Resuscitation Post-Cardiac Surgery: A Randomized Controlled Trial. J Cardiovasc Dev Dis 2023 Feb 4;10(2):67. PMID:36826563

6. Moll-Khosrawi P, Falb A, Pinnschmidt H, Zöllner C, Issleib M. Virtual reality as a teaching method for resuscitation training in undergraduate first year medical students during COVID-19 pandemic: a randomised controlled trial. BMC Med Educ 2022 Jun 22;22(1):483. PMID:35733135

7. Rochlen LR, Putnam E, Levine R, Tait AR. Mixed reality simulation for peripheral intravenous catheter placement training. BMC Med Educ 2022 Dec 17;22(1):876. PMID:36528576

8. White WW, Jung MJ. Three-Dimensional Virtual Reality Spinal Cord Stimulator Training Improves Trainee Procedural Confidence and Performance. Neuromodulation 2022 May 12;S1094-7159(22)00631–6. PMID:35570148

9. Issleib M, Kromer A, Pinnschmidt HO, Süss-Havemann C, Kubitz JC. Virtual reality as a teaching method for resuscitation training in undergraduate first year medical students: a randomized

controlled trial. Scand J Trauma Resusc Emerg Med 2021 Dec;29(1):27. doi: 10.1186/s13049-021-00836-y

10. Lee DK, Im CW, Jo YH, Chang T, Song JL, Luu C, Mackinnon R, Pillai S, Lee CN, Jheon S, Ahn S, Won SH. Comparison of extended reality and conventional methods of basic life support training: protocol for a multinational, pragmatic, noninferiority, randomised clinical trial (XR BLS trial). Trials 2021 Dec 20;22(1):946. PMID:34930418

11. Mah E, Yu J, Deck M, Lyster K, Kawchuk J, Turnquist A, Thoma B. Immersive Video Modeling Versus Traditional Video Modeling for Teaching Central Venous Catheter Insertion to Medical Residents. Cureus 2021 Mar 2;13(3):e13661. PMID:33824812

12. Bube S, Dagnaes-Hansen J, Mahmood O, Rohrsted M, Bjerrum F, Salling L, Hansen RB, Konge L. Simulation-based training for flexible cystoscopy - A randomized trial comparing two approaches. Heliyon 2020 Jan;6(1):e03086. PMID:31922043

13. Nas J, Thannhauser J, Vart P, van Geuns R-J, Muijsers HEC, Mol J-Q, Aarts GWA, Konijnenberg LSF, Gommans DHF, Ahoud-Schoenmakers SGAM, Vos JL, van Royen N, Bonnes JL, Brouwer MA. Effect of Face-to-Face vs Virtual Reality Training on Cardiopulmonary Resuscitation Quality: A Randomized Clinical Trial. JAMA Cardiol 2020 Mar 1;5(3):328–335. PMID:31734702

14. Ameri G, Rankin A, Baxter JSH, Moore J, Ganapathy S, Peters TM, Chen ECS. Development and Evaluation of an Augmented Reality Ultrasound Guidance System for Spinal Anesthesia: Preliminary Results. Ultrasound in Medicine & Biology 2019 Oct;45(10):2736–2746. doi: 10.1016/j.ultrasmedbio.2019.04.026

15. Sappenfield J, Grek S, Cooper LA, Lizdas DE, Lampotang S. Reduced Complications of Supraclavicular Approach in Simulated Central Venous Access: Applicability to Military Medicine. Military Medicine 2019 Mar 1;184(Supplement_1):329–334. doi: 10.1093/milmed/usy381

16. Aebersold M, Voepel-Lewis T, Cherara L, Weber M, Khouri C, Levine R, Tait AR. Interactive Anatomy-Augmented Virtual Simulation Training. Clin Simul Nurs 2018 Feb;15:34–41. PMID:29861797

17. Casso G, Schoettker P, Savoldelli GL, Azzola A, Cassina T. Development and Initial Evaluation of a Novel, Ultraportable, Virtual Reality Bronchoscopy Simulator: The Computer Airway Simulation System. Anesth Analg 2019 Nov;129(5):1258–1264. doi: 10.1213/ANE.0000000000003316

18. Jensen JK, Dyre L, Jørgensen ME, Andreasen LA, Tolsgaard MG. Simulation-based point-of-care ultrasound training: a matter of competency rather than volume. Acta Anaesthesiol Scand 2018 Jul;62(6):811–819. PMID:29392718

19. Jiang B, Ju H, Zhao Y, Yao L, Feng Y. Comparison of the Efficacy and Efficiency of the Use of Virtual Reality Simulation With High-Fidelity Mannequins for Simulation-Based Training of Fiberoptic Bronchoscope Manipulation. Sim Healthcare 2018 Apr;13(2):83–87. PMID:29621098

20. Wong DT, Mehta A, Singh KP, Leong SM, Ooi A, Niazi A, You-Ten E, Okrainec A, Patel R, Singh M, Wong J. The effect of virtual reality bronchoscopy simulator training on performance of

bronchoscopic-guided intubation in patients: A randomised controlled trial. European Journal of Anaesthesiology 2019 Mar;36(3):227–233. doi: 10.1097/EJA.0000000000000890

21. Rochlen LR, Levine R, Tait AR. First-Person Point-of-View–Augmented Reality for Central Line Insertion Training: A Usability and Feasibility Study. Sim Healthcare 2017 Feb;12(1):57–62. doi: 10.1097/SIH.0000000000000185

22. Mahmood F, Mahmood E, Dorfman RG, Mitchell J, Mahmood F-U, Jones SB, Matyal R. Augmented Reality and Ultrasound Education: Initial Experience. J Cardiothorac Vasc Anesth 2018 Jun;32(3):1363–1367. PMID:29452879

23. Latif R, Bautista A, Duan X, Neamtu A, Wu D, Wadhwa A, Akça O. Teaching basic fiberoptic intubation skills in a simulator: initial learning and skills decay. J Anesth 2016 Feb;30(1):12–19. PMID:26493397

24. Nilsson PM, Russell L, Ringsted C, Hertz P, Konge L. Simulation-based training in flexible fibreoptic intubation: A randomised study. Eur J Anaesthesiol 2015 Sep;32(9):609–614. PMID:24809483

25. Failor E, Bowdle A, Jelacic S, Togashi K. High-fidelity simulation of lung isolation with double-lumen endotracheal tubes and bronchial blockers in anesthesiology resident training. J Cardiothorac Vasc Anesth 2014 Aug;28(4):865–869. PMID:24231196

26. O'Sullivan O, Iohom G, O'Donnell BD, Shorten GD. The effect of simulation-based training on initial performance of ultrasound-guided axillary brachial plexus blockade in a clinical setting - a pilot study. BMC Anesthesiol 2014;14:110. PMID:25844062

27. Robinson AR, Gravenstein N, Cooper LA, Lizdas D, Luria I, Lampotang S. A Mixed-Reality Part-Task Trainer for Subclavian Venous Access. Simulation in Healthcare: The Journal of the Society for Simulation in Healthcare 2014 Feb;9(1):56–64. doi: 10.1097/SIH.0b013e31829b3fb3

28. De Oliveira GS, Glassenberg R, Chang R, Fitzgerald P, McCarthy RJ. Virtual airway simulation to improve dexterity among novices performing fibreoptic intubation. Anaesthesia 2013 Oct;68(10):1053–1058. PMID:23952805

29. Kulcsár Z, O'Mahony E, Lövquist E, Aboulafia A, Šabova D, Ghori K, Iohom G, Shorten G. Preliminary evaluation of a virtual reality-based simulator for learning spinal anesthesia. Journal of Clinical Anesthesia 2013 Mar;25(2):98–105. doi: 10.1016/j.jclinane.2012.06.015

30. Grottke O, Ntouba A, Ullrich S, Liao W, Fried E, Prescher A, Deserno TM, Kuhlen T, Rossaint R. Virtual reality-based simulator for training in regional anaesthesia. Br J Anaesth 2009 Oct;103(4):594–600. PMID:19706630

31. Goldmann K, Steinfeldt T. Acquisition of basic fiberoptic intubation skills with a virtual reality airway simulator. J Clin Anesth 2006 May;18(3):173–178. PMID:16731318

32. Reznek MA, Rawn CL, Krummel TM. Evaluation of the educational effectiveness of a virtual reality intravenous insertion simulator. Acad Emerg Med 2002 Nov;9(11):1319–1325. PMID:12414488

33. Hoek KB, van Velzen M, Sarton EY. Patient-embodied virtual reality as a learning tool for therapeutic communication skills among anaesthesiologists: A phenomenological study. Patient Educ Couns 2023 May 6;114:107789. PMID:37230038

34. Knudsen MH, Breindahl N, Dalsgaard T-S, Isbye D, Mølbak AG, Tiwald G, Svendsen MBS, Konge L, Bergström J, Todsen T. Using Virtual Reality Head-Mounted Displays to Assess Skills in Emergency Medicine: Validity Study. J Med Internet Res 2023 Jun 6;25:e45210. PMID:37279049

35. Liaw SY, Sutini null, Chua WL, Tan JZ, Levett-Jones T, Ashokka B, Te Pan TL, Lau ST, Ignacio J. Desktop Virtual Reality Versus Face-to-Face Simulation for Team-Training on Stress Levels and Performance in Clinical Deterioration: a Randomised Controlled Trial. J Gen Intern Med 2023 Jan;38(1):67–73. PMID:35501626

36. Speidel R, Schneider A, Walter S, Grab-Kroll C, Oechsner W. Immersive medium for early clinical exposure - knowledge acquisition, spatial orientation and the unexpected role of annotation in 360° VR photos. GMS J Med Educ 2023;40(1):Doc8. PMID:36923314

37. Hess O, Qian J, Bruce J, Wang E, Rodriguez S, Haber N, Caruso TJ. Communication Skills Training Using Remote Augmented Reality Medical Simulation: a Feasibility and Acceptability Qualitative Study. Med Sci Educ 2022 Oct;32(5):1005–1014. PMID:35966166

38. Truong H, Qi D, Ryason A, Sullivan AM, Cudmore J, Alfred S, Jones SB, Parra JM, De S, Jones DB. Does your team know how to respond safely to an operating room fire? Outcomes of a virtual reality, AI-enhanced simulation training. Surg Endosc 2022 May;36(5):3059–3067. PMID:34264400

39. Chheang V, Fischer V, Buggenhagen H, Huber T, Huettl F, Kneist W, Preim B, Saalfeld P, Hansen C. Toward interprofessional team training for surgeons and anesthesiologists using virtual reality. Int J Comput Assist Radiol Surg 2020 Dec;15(12):2109–2118. PMID:33083969

40. Katz D, Shah R, Kim E, Park C, Shah A, Levine A, Burnett G. Utilization of a Voice-Based Virtual Reality Advanced Cardiac Life Support Team Leader Refresher: Prospective Observational Study. J Med Internet Res 2020 Mar 12;22(3):e17425. PMID:32163038

41. Lerner D, Mohr S, Schild J, Göring M, Luiz T. An Immersive Multi-User Virtual Reality for Emergency Simulation Training: Usability Study. JMIR Serious Games 2020 Jul 31;8(3):e18822. doi: 10.2196/18822

42. Masson C, Birgand G, Castro-Sánchez E, Eichel VM, Comte A, Terrisse H, Rubens-Duval B, Gillois P, Albaladejo P, Picard J, Bosson JL, Mutters NT, Landelle C. Is virtual reality effective to teach prevention of surgical site infections in the operating room? study protocol for a randomised controlled multicentre trial entitled VIP Room study. BMJ Open 2020 Jun;10(6):e037299. doi: 10.1136/bmjopen-2020-037299

43. Wunder L, Gomez NAG, Gonzalez JE, Mitzova-Vladinov G, Cacchione M, Mato J, Foronda CL, Groom JA. Fire in the Operating Room: Use of Mixed Reality Simulation with Nurse Anesthesia Students. Informatics 2020 Sep 30;7(4):40. doi: 10.3390/informatics7040040

44. Abbas S, Chan V, Jin R, Kim E, Lorello G, Niazi A, Singh M, Unger Z. ESRA19-0614 Virtual reality simulation for teaching and assessment of anesthesia crisis management. Late Breakers BMJ Publishing Group Ltd; 2019. p. A262–A262. doi: 10.1136/rapm-2019-ESRAABS2019.480

45. Erlinger LR, Bartlett A, Perez A. High-Fidelity Mannequin Simulation versus Virtual Simulation for Recognition of Critical Events by Student Registered Nurse Anesthetists. AANA J 2019 Apr;87(2):105–109. PMID:31587722

46. Semeraro F, Ristagno G, Giulini G, Kayal JS, Cavallo P, Farabegoli L, Tucci R, Scelsi S, Grieco NB, Scapigliati A. Back to reality: A new blended pilot course of Basic Life Support with Virtual Reality. Resuscitation 2019 May;138:18–19. PMID:30844415

47. Sankaranarayanan G, Wooley L, Hogg D, Dorozhkin D, Olasky J, Chauhan S, Fleshman JW, De S, Scott D, Jones DB. Immersive virtual reality-based training improves response in a simulated operating room fire scenario. Surg Endosc 2018 Aug;32(8):3439–3449. PMID:29372313

48. Cordar A, Wendling A, White C, Lampotang S, Lok B. Repeat after me: Using mixed reality humans to influence best communication practices. 2017 IEEE Virtual Reality (VR) Los Angeles, CA, USA: IEEE; 2017. p. 148–156. doi: 10.1109/VR.2017.7892242

49. Dorozhkin D, Olasky J, Jones DB, Schwaitzberg SD, Jones SB, Cao CGL, Molina M, Henriques S, Wang J, Flinn J, De S, SAGES FUSE Committee. OR fire virtual training simulator: design and face validity. Surg Endosc 2017 Sep;31(9):3527–3533. PMID:28039649

50. Real FJ, DeBlasio D, Beck AF, Ollberding NJ, Davis D, Cruse B, Samaan Z, McLinden D, Klein MD. A Virtual Reality Curriculum for Pediatric Residents Decreases Rates of Influenza Vaccine Refusal. Acad Pediatr 2017 Jun;17(4):431–435. PMID:28126612

51. Cordar A, Robb A, Wendling A, Lampotang S, White C, Lok B. Virtual Role-Models: Using Virtual Humans to Train Best Communication Practices for Healthcare Teams. In: Brinkman W-P, Broekens J, Heylen D, editors. Intelligent Virtual Agents Cham: Springer International Publishing; 2015. p. 229–238. doi: 10.1007/978-3-319-21996-7_23

52. Baytar A, Bollucuo Lu K. Effect of virtual reality on preoperative anxiety in patients undergoing septorhinoplasty. Braz J Anesthesiol 2023;73(2):159–164. PMID:34562488

53. Kim Y, Yoo SH, Chun J, Kim J-H, Youn YH, Park H. Relieving Anxiety Through Virtual Reality Prior to Endoscopic Procedures. Yonsei Med J 2023 Feb;64(2):117–122. PMID:36719019

54. Le Du K, Septans A-L, Maloisel F, Vanquaethem H, Schmitt A, Le Goff M, Clavert A, Zinger M, Bourgeois H, Dupuis O, Denis F, Bouchard S. A New Option for Pain Prevention Using a Therapeutic Virtual Reality Solution for Bone Marrow Biopsy (REVEH Trial): Open-Label, Randomized, Multicenter, Phase 3 Study. J Med Internet Res 2023 Feb 15;25:e38619. PMID:36790852

55. Lind A, Ahsan M, Totzeck M, Al-Rashid F, Haddad A, Dubler S, Brenner T, Skarabis A, El Gabry M, Rassaf T, Jánosi RA. Virtual reality-assisted distraction during transcatheter aortic valve implantation under local anaesthesia: A randomised study. Int J Cardiol 2023 Sep 15;387:131130. PMID:37355243

56. Lopes K, Dessieux T, Rousseau C, Beloeil H. Virtual Reality as a Hypnotic Tool in the Management of Anxiety During the Performance of the Axillary Block. J Med Syst 2023 Mar 1;47(1):31. PMID:36856907

57. Pelazas-Hernández JA, Varillas-Delgado D, González-Casado T, Cristóbal-Quevedo I, Alonso-Bermejo A, Ronchas-Martínez M, Cristóbal-García I. The Effect of Virtual Reality on the Reduction of Pain in Women with an Indication for Outpatient Diagnostic Hysteroscopy: A Randomized Controlled Trial. J Clin Med 2023 May 24;12(11):3645. PMID:37297840

58. Barry KS, Nimma SR, Spaulding AC, Wilke BK, Torp KD, Ledford CK. Perioperative Outcomes of Immersive Virtual Reality as Adjunct Anesthesia in Primary Total Hip and Knee Arthroplasty. Arthroplast Today 2022 Dec;18:84–88. PMID:36312886

59. Boonreunya N, Nopawong E, Yongsiriwit K, Chirapongsathorn S. Virtual reality distraction during upper gastrointestinal endoscopy: a randomized controlled trial. J Gastroenterol Hepatol 2022 May;37(5):855–860. PMID:35080055

60. Faruki AA, Nguyen TB, Gasangwa D-V, Levy N, Proeschel S, Yu J, Ip V, McGourty M, Korsunsky G, Novack V, Mueller AL, Banner-Goodspeed V, Rozental TD, O'Gara BP. Virtual reality immersion compared to monitored anesthesia care for hand surgery: A randomized controlled trial. PLoS One 2022;17(9):e0272030. PMID:36129891

61. Fouks Y, Kern G, Cohen A, Reicher L, Shapira Z, Many A, Yogev Y, Rattan G. A virtual reality system for pain and anxiety management during outpatient hysteroscopy—A randomized control trial. European Journal of Pain 2022 Mar;26(3):600–609. doi: 10.1002/ejp.1882

62. Goergen DI, Freitas DMDO. Virtual Reality as a distraction therapy during cystoscopy: a clinical trial. Rev Col Bras Cir 2022;49:e20223138. PMID:35584530

63. Krish G, Immerman I, Kinjo S. Virtual reality may reduce anxiety and enhance surgical experience during wide-awake local anaesthesia no tourniquet surgery: A report of two cases. J Perioper Pract 2022 Jun;32(6):136–141. PMID:34190639

64. Lame C, Mougin J, Postiglione TJ, Fabre D, Haulon S. Virtual Reality to Ease Endovascular Repair of Thoracoabdominal Aneurysms Under Local Anesthesia. J Endovasc Ther 2023 Apr;30(2):312–315. PMID:35184605

65. Rousseaux F, Dardenne N, Massion PB, Ledoux D, Bicego A, Donneau A-F, Faymonville M-E, Nyssen A-S, Vanhaudenhuyse A. Virtual reality and hypnosis for anxiety and pain management in intensive care units: A prospective randomised trial among cardiac surgery patients. Eur J Anaesthesiol 2022 Jan 1;39(1):58–66. PMID:34783683

66. Soret L, Gendron N, Rivet N, Chocron R, Macraigne L, Clausse D, Cholley B, Gaussem P, Smadja DM, Darnige L. Pain Assessment Using Virtual Reality Facemask During Bone Marrow Aspiration: Prospective Study Including Propensity-Matched Analysis. JMIR Serious Games 2022 Oct 12;10(4):e33221. PMID:36222814

67. Bozdoğan Yeşilot S, Ciftci H, Yener MK. Using a Virtual Reality and Communication Intervention to Reduce Pain and Anxiety in Patients Undergoing Lipoma Excision With Local Anesthesia: A Randomized Controlled Trial. AORN J 2022 May;115(5):437–449. PMID:35476192

68. Karaman D, Taşdemir N. The Effect of Using Virtual Reality During Breast Biopsy on Pain and Anxiety: A Randomized Controlled Trial. J Perianesth Nurs 2021 Dec;36(6):702–705. PMID:34686402

69. Ledford CK, VanWagner MJ, Sherman CE, Torp KD. Immersive Virtual Reality Used as Adjunct Anesthesia for Conversion Total Hip Arthroplasty in a 100-Year-Old Patient. Arthroplast Today 2021 Aug;10:149–153. PMID:34401418

70. Łuczak M, Nowak Ł, Chorbińska J, Galik K, Kiełb P, Łaszkiewicz J, Tukiendorf A, Kościelska-Kasprzak K, Małkiewicz B, Zdrojowy R, Szydełko T, Krajewski W. Influence of Virtual Reality Devices on Pain and Anxiety in Patients Undergoing Cystoscopy Performed under Local Anaesthesia. J Pers Med 2021 Nov 16;11(11):1214. PMID:34834565

71. Peuchot H, Khakha R, Riera V, Ollivier M, Argenson J-N. Intraoperative virtual reality distraction in TKA under spinal anesthesia: a preliminary study. Arch Orthop Trauma Surg 2021 Dec;141(12):2323–2328. doi: 10.1007/s00402-021-04065-x

72. Roxburgh T, Li A, Guenancia C, Pernollet P, Bouleti C, Alos B, Gras M, Kerforne T, Frasca D, Le Gal F, Christiaens L, Degand B, Garcia R. Virtual Reality for Sedation During Atrial Fibrillation Ablation in Clinical Practice: Observational Study. J Med Internet Res 2021 May 27;23(5):e26349. doi: 10.2196/26349

73. Tharion JG, Kale S. Patient Satisfaction Through an Immersive Experience Using a Mobile Phone-Based Head-Mounted Display During Arthroscopic Knee Surgery Under Spinal Anesthesia: A Randomized Clinical Trial. Anesth Analg 2021 Oct 1;133(4):940–948. PMID:34283040

74. Touil N, Pavlopoulou A, Momeni M, Van Pee B, Barbier O, Sermeus L, Roelants F. Evaluation of virtual reality combining music and a hypnosis session to reduce anxiety before hand surgery under axillary plexus block: A prospective study. Int J Clin Pract 2021 Dec;75(12):e15008. PMID:34811860

75. Turrado V, Guzmán Y, Jiménez-Lillo J, Villegas E, de Lacy FB, Blanch J, Balibrea JM, Lacy A. Exposure to virtual reality as a tool to reduce peri-operative anxiety in patients undergoing colorectal cancer surgery: a single-center prospective randomized clinical trial. Surg Endosc 2021 Jul;35(7):4042–4047. PMID:33683433

76. Vogt L, Klasen M, Rossaint R, Goeretz U, Ebus P, Sopka S. Virtual Reality Tour to Reduce Perioperative Anxiety in an Operating Setting Before Anesthesia: Randomized Clinical Trial. J Med Internet Res 2021 Sep 1;23(9):e28018. PMID:34252034

77. Alaterre C, Duceau B, Sung Tsai E, Zriouel S, Bonnet F, Lescot T, Verdonk F. Virtual Reality for PEripheral Regional Anesthesia (VR-PERLA Study). JCM 2020 Jan 13;9(1):215. doi: 10.3390/jcm9010215

78. Chan JJI, Yeam CT, Kee HM, Tan CW, Sultana R, Sia ATH, Sng BL. The use of pre-operative virtual reality to reduce anxiety in women undergoing gynecological surgeries: a prospective cohort study. BMC Anesthesiol 2020 Dec;20(1):261. doi: 10.1186/s12871-020-01177-6

79. Huang MY, Scharf S, Chan PY. Effects of immersive virtual reality therapy on intravenous patient-controlled sedation during orthopaedic surgery under regional anesthesia: A randomized controlled trial. Scherag A, editor. PLoS ONE 2020 Feb 24;15(2):e0229320. doi: 10.1371/journal.pone.0229320

80. Kist M, Bekemeyer Z, Ralls L, Carvalho B, Rodriguez ST, Caruso TJ. Virtual reality successfully provides anxiolysis to laboring women undergoing epidural placement. Journal of Clinical Anesthesia 2020 May;61:109635. doi: 10.1016/j.jclinane.2019.109635

81. Sridhar A, Shiliang Z, Woodson R, Kwan L. Non-pharmacological anxiety reduction with immersive virtual reality for first-trimester dilation and curettage: a pilot study. Eur J Contracept Reprod Health Care 2020 Dec;25(6):480–483. PMID:33140989

82. Faruki A, Nguyen T, Proeschel S, Levy N, Yu J, Ip V, Mueller A, Banner-Goodspeed V, O’Gara B. Virtual reality as an adjunct to anesthesia in the operating room. Trials 2019 Dec 27;20(1):782. PMID:31882015

83. Hoxhallari E, Behr IJ, Bradshaw JS, Morkos MS, Haan PS, Schaefer MC, Clarkson JHW. Virtual Reality Improves the Patient Experience during Wide-Awake Local Anesthesia No Tourniquet Hand Surgery: A Single-Blind, Randomized, Prospective Study. Plastic and Reconstructive Surgery 2019 Aug;144(2):408–414. doi: 10.1097/PRS.0000000000005831

84. Moon JY, Shin J, Chung J, Ji S-H, Ro S, Kim WH. Virtual Reality Distraction during Endoscopic Urologic Surgery under Spinal Anesthesia: A Randomized Controlled Trial. J Clin Med 2018 Dec 20;8(1):2. PMID:30577461

85. Thomas JJ, Albietz J, Polaner D. Virtual reality for lumbar puncture in a morbidly obese patient with leukemia. Paediatr Anaesth 2018 Nov;28(11):1059–1060. PMID:30284748

86. Chan PY, Scharf S. Virtual Reality as an Adjunctive Nonpharmacological Sedative During Orthopedic Surgery Under Regional Anesthesia: A Pilot and Feasibility Study. Anesth Analg 2017 Oct;125(4):1200–1202. PMID:28598921

87. Mosso Vázquez JL, Wiederhold BK, Miller I, Wiederhold MD. Virtual Reality Assisted Anaesthesia During Upper Gastrointestinal Endoscopy: Report of 115 Cases. EMJ Innov 2017 Jan 10;75–82. doi: 10.33590/emjinnov/10314545

88. Mosso Vázquez J, Wiederhold B, Miller I, Lara D, Wiederhold M. Virtual Reality Assisted Anesthesia (VRAA) during Upper Gastrointestinal Endoscopy: Report of 115 Cases— Analysis of Physiological Responses. Surgical Research Updates 2017 Mar 6;5. doi: 10.12970/2311-9888.2017.05.01

89. Pandya PG, Kim TE, Howard SK, Stary E, Leng JC, Hunter OO, Mariano ER. Virtual reality distraction decreases routine intravenous sedation and procedure-related pain during

preoperative adductor canal catheter insertion: a retrospective study. Korean J Anesthesiol 2017;70(4):439. PMID:28794840

90. Bekelis K, Calnan D, Simmons N, MacKenzie TA, Kakoulides G. Effect of an Immersive Preoperative Virtual Reality Experience on Patient Reported Outcomes: A Randomized Controlled Trial. Ann Surg 2017 Jun;265(6):1068–1073. PMID:27906757

91. Jahani Shoorab N, Ebrahimzadeh Zagami S, Nahvi A, Mazluom SR, Golmakani N, Talebi M, Pabarja F. The Effect of Virtual Reality on Pain in Primiparity Women during Episiotomy Repair: A Randomize Clinical Trial. Iran J Med Sci 2015 May;40(3):219–224. PMID:25999621

92. Mosso Vázquez JL, Gorini A, De La Cerda G, Obrador T, Almazan A, Mosso D, Nieto JJ, Riva G. Virtual reality on mobile phones to reduce anxiety in outpatient surgery. Stud Health Technol Inform 2009;142:195–200. PMID:19377147

93. Bergauer L, Akbas S, Braun J, Ganter MT, Meybohm P, Hottenrott S, Zacharowski K, Raimann FJ, Rivas E, López-Baamonde M, Spahn DR, Noethiger CB, Tscholl DW, Roche TR. Visual Blood, Visualisation of Blood Gas Analysis in Virtual Reality, Leads to More Correct Diagnoses: A Computer-Based, Multicentre, Simulation Study. Bioengineering (Basel) 2023 Mar 8;10(3):340. PMID:36978731

94. Berger A, Choudhry OJ, Kondziolka D. Augmented Reality-Assisted Percutaneous Rhizotomy for Trigeminal Neuralgia. Oper Neurosurg (Hagerstown) 2023 Jun 1;24(6):665–669. PMID:36815787

95. Hayasaka T, Kawano K, Onodera Y, Suzuki H, Nakane M, Kanoto M, Kawamae K. Comparison of accuracy between augmented reality/mixed reality techniques and conventional techniques for epidural anesthesia using a practice phantom model kit. BMC Anesthesiol 2023 May 20;23(1):171. PMID:37210521

96. Jun EK, Lim S, Seo J, Lee KH, Lee JH, Lee D, Koh JC. Augmented Reality-Assisted Navigation System for Transforaminal Epidural Injection. J Pain Res 2023;16:921–931. PMID:36960464

97. Reinacher PC, Cimniak A, Demerath T, Schallner N. Usage of augmented reality for interventional neuraxial procedures: A phantom-based study. Eur J Anaesthesiol 2023 Feb 1;40(2):121–129. PMID:36121289

98. Iliff HA, Ahmad I, Evans S, Ingham J, Rees G, Woodford C. Using virtual reality for difficult airway management planning. Anaesth Rep 2022;10(2):e12175. PMID:35813404

99. Moon RDC, Barua NU. Usability of mixed reality in awake craniotomy planning. Br J Neurosurg 2022 Dec 20;1–5. PMID:36537230

100. Seong H, Yun D, Yoon KS, Kwak JS, Koh JC. Development of pre-procedure virtual simulation for challenging interventional procedures: an experimental study with clinical application. Korean J Pain 2022 Oct 1;35(4):403–412. PMID:36175339

101. Tanwani J, Alam F, Matava C, Choi S, McHardy P, Singer O, Cheong G, Wiegelmann J. Development of a Head-Mounted Holographic Needle Guidance System for Enhanced

Ultrasound-Guided Neuraxial Anesthesia: System Development and Observational Evaluation. JMIR Form Res 2022 Jun 23;6(6):e36931. PMID:35737430

102. Follmann A, Ruhl A, Gösch M, Felzen M, Rossaint R, Czaplik M. Augmented Reality for Guideline Presentation in Medicine: Randomized Crossover Simulation Trial for Technically Assisted Decision-making. JMIR Mhealth Uhealth 2021 Oct 18;9(10):e17472. PMID:34661548

103. Romare C, Enlöf P, Anderberg P, Jildenstål P, Sanmartin Berglund J, Skär L. Nurse anesthetists' experiences using smart glasses to monitor patients' vital signs during anesthesia care: A qualitative study. PLoS One 2021;16(4):e0250122. PMID:33882100

104. Schlosser PD, Grundgeiger T, Sanderson PM, Happel O. An exploratory clinical evaluation of a head-worn display based multiple-patient monitoring application: impact on supervising anesthesiologists' situation awareness. J Clin Monit Comput 2019 Dec;33(6):1119–1127. doi: 10.1007/s10877-019-00265-4

105. Hetherington J, Lessoway V, Gunka V, Abolmaesumi P, Rohling R. SLIDE: automatic spine level identification system using a deep convolutional neural network. Int J Comput Assist Radiol Surg 2017 Jul;12(7):1189–1198. PMID:28361323

106. Kaneko N, Tsunoda M, Mitsuhashi M, Okubo K, Takeshima T, Sehara Y, Nagai M, Kawai K. Ultrasound-Guided Fine-Needle Aspiration in the Neck Region Using an Optical See-Through Head-Mounted Display: A Randomized Controlled Trial. J Ultrasound Med 2017 Oct;36(10):2071–2077. PMID:28504313

107. Kaneko N, Sato M, Takeshima T, Sehara Y, Watanabe E. Ultrasound-guided central venous catheterization using an optical see-through head-mounted display: A pilot study. J Clin Ultrasound 2016 Oct;44(8):487–491. PMID:27297681

108. Przkora R, McGrady W, Vasilopoulos T, Gravenstein N, Solanki D. Evaluation of the Head-Mounted Display for Ultrasound-Guided Peripheral Nerve Blocks in Simulated Regional Anesthesia. Pain Med 2015 Nov;16(11):2192–2194. PMID:25930716

109. Ashab HA-D, Lessoway VA, Khallaghi S, Cheng A, Rohling R, Abolmaesumi P. AREA: an augmented reality system for epidural anaesthesia. Annu Int Conf IEEE Eng Med Biol Soc 2012;2012:2659–2663. PMID:23366472

110. Udani AD, Harrison TK, Howard SK, Kim TE, Brock-Utne JG, Gaba DM, Mariano ER. Preliminary study of ergonomic behavior during simulated ultrasound-guided regional anesthesia using a head-mounted display. J Ultrasound Med 2012 Aug;31(8):1277–1280. PMID:22837293

111. Liu D, Jenkins SA, Sanderson PM, Watson MO, Leane T, Kruys A, Russell WJ. Monitoring with head-mounted displays: performance and safety in a full-scale simulator and part-task trainer. Anesth Analg 2009 Oct;109(4):1135–1146. PMID:19762741

112. Sanderson PM, Watson MO, Russell WJ, Jenkins S, Liu D, Green N, Llewelyn K, Cole P, Shek V, Krupenia SS. Advanced auditory displays and head-mounted displays: advantages and disadvantages for monitoring by the distracted anesthesiologist. Anesth Analg 2008 Jun;106(6):1787–1797. PMID:18499611

113. Ormerod DF, Ross B, Naluai-Cecchini A. Use of an augmented reality display of patient monitoring data to enhance anesthesiologists' response to abnormal clinical events. Stud Health Technol Inform 2003;94:248–250. PMID:15455902

114. Block FE, Yablok DO, McDonald JS. Clinical evaluation of the "head-up" display of anesthesia data. Preliminary communication. Int J Clin Monit Comput 1995 Feb;12(1):21–24. PMID:7782663
